# YAP/TAZ link cell mechanics to Notch signalling to control epidermal stem cell fate

**DOI:** 10.1038/ncomms15206

**Published:** 2017-05-17

**Authors:** Antonio Totaro, Martina Castellan, Giusy Battilana, Francesca Zanconato, Luca Azzolin, Stefano Giulitti, Michelangelo Cordenonsi, Stefano Piccolo

**Affiliations:** 1 Department of Molecular Medicine (DMM), University of Padua School of Medicine, viale Colombo 3, Padua 35126, Italy; 2 Department of Industrial Engineering (DII), University of Padua, via Marzolo 9, Padua 35131, Italy

## Abstract

How the behaviour of somatic stem cells (SCs) is influenced by mechanical signals remains a black-box in cell biology. Here we show that YAP/TAZ regulation by cell shape and rigidity of the extracellular matrix (ECM) dictates a pivotal SC decision: to remain undifferentiated and grow, or to activate a terminal differentiation programme. Notably, mechano-activation of YAP/TAZ promotes epidermal stemness by inhibition of Notch signalling, a key factor for epidermal differentiation. Conversely, YAP/TAZ inhibition by low mechanical forces induces Notch signalling and loss of SC traits. As such, mechano-dependent regulation of YAP/TAZ reflects into mechano-dependent regulation of Notch signalling. Mechanistically, at least in part, this is mediated by YAP/TAZ binding to distant enhancers activating the expression of Delta-like ligands, serving as ‘in *cis*' inhibitors of Notch. Thus YAP/TAZ mechanotransduction integrates with cell–cell communication pathways for fine-grained orchestration of SC decisions.

## Abstract (web summary)

Notch signalling is a fundamental negative regulator of epidermal stemness. Here, the authors show that cell mechanics through YAP/TAZ activity prevent primary human keratinocytes from differentiating by inhibiting cell-autonomous Notch signals.

Cell behaviour is profoundly influenced by the physical and architectural features of the cell microenvironment^1^. These features template cell–cell and cell–extracellular matrix (ECM) adhesion sites that provide the mechanical signals to control the shape of the cell, define its localization and spatial relationships with other cells in tissues and, ultimately, regulate cell fate^2^,^3^,^4^,^5^,^6^. The epidermis is a point in case: cell attachment to the basement membrane is associated to changes in cytoskeletal rigidity and organization that preserve stemness and proliferative capacity and inhibit differentiation. As cells lose contact with the basement membrane and enter into the suprabasal layers of this stratified epithelium, they initiate a terminal differentiation programme until they are shed from the tissue surface^7^,^8^,^9^.

The epidermis is a paradigm mechanosensitive tissue: upon stretching—such as during postnatal development or pregnancy—it adapts to body growth by increasing its own size through an exhalted proliferation of the basal layer. Wounding is also associated with increased rigidity of the underlying dermis and this is perceived by basal keratinocytes at the wound's margin to initiate migration, proliferation and wound re-epithelialization^10^.

Such mechano-dependent control of epidermal cell fate can be at least in part recapitulated *in vitro* by culturing epidermal progenitor cells into engineered surfaces: when these cells are cultured over a rigid ECM, they adopt a spread shape and preserve their undifferentiated, stem cell (SC)-like state; however, if they are forced to adhere to small adhesive areas or to a soft ECM, they round-up and permanently exit cell cycle and differentiate^11^,^12^,^13^,^14^,^15^. Little is known, however, on the causal relationships between cell shape and fate and on the transcription factors transducing biomechanical signals to epidermal SCs. Here we have investigated the role of YAP and TAZ in these events.

YAP/TAZ control organ size during embryonic development possibly by triggering amplification of progenitors of several tissues, including the epidermis^16^,^17^,^18^,^19^,^20^. YAP/TAZ are also essential transducers of mechanical signals in a number of cellular contexts^21^,^22^,^23^. YAP/TAZ are active in cells experiencing a rigid ECM, a spread cell shape and a tense cytoskeleton and are turned off by softer ECM environments or attachment to small adhesive areas^24^. Here we found that mechanical regulation of YAP/TAZ in epidermal progenitors represents a mechanism by which the structural and physical traits of the tissue environment may imbue SC fate decisions.

This study also brought us to explore how mechanical regulation of YAP/TAZ may control other short-range signalling interactions by which neighbouring cells mutually regulate and refine each other's fate. In the epidermis, the paradigm of this communication is Notch signalling: Notch activation is critical to promote the differentiated state suprabasally, while basal cells must be somehow protected from this cascade^25^,^26^,^27^.

The contrasting effects of YAP/TAZ and Notch signalling in epidermal cell fate have not been connected before. Here we find that mechanical signals use YAP/TAZ to control Notch signalling: YAP/TAZ transcriptionally regulate the expression of Notch inhibitors, such as the epidermal SC factor DLL1, known for blocking Notch signalling ‘in *cis*', thus protecting the undifferentiated state^28^,^29^,^30^. Thus YAP/TAZ mechanotransduction integrates physical and architectural signals with Notch regulation, such as coordinating mechanosensitive pathways, cell–cell communication and SC preservation versus differentiation.

## Results

### YAP/TAZ mechanotransduction controls epidermal SC fate

We initiated this study by investigating how mechanical signals regulate the choice of differentiation versus proliferation of epidermal progenitor cells through changes in YAP/TAZ activity. For this, we used primary neonatal human keratinocytes expanded *in vitro* for few passages to obtain a culture in rapid growth phase (see Supplementary Fig. 1a). These cultures are highly enriched of epidermal SCs, as about 90% of these cells displayed elevated expression of p63, as detected by immunofluorescence (IF; Supplementary Fig. 1b)^31^, and of β1 integrin, as determined by flow cytometry (Supplementary Fig. 1c)^32^.

**Figure 1:**
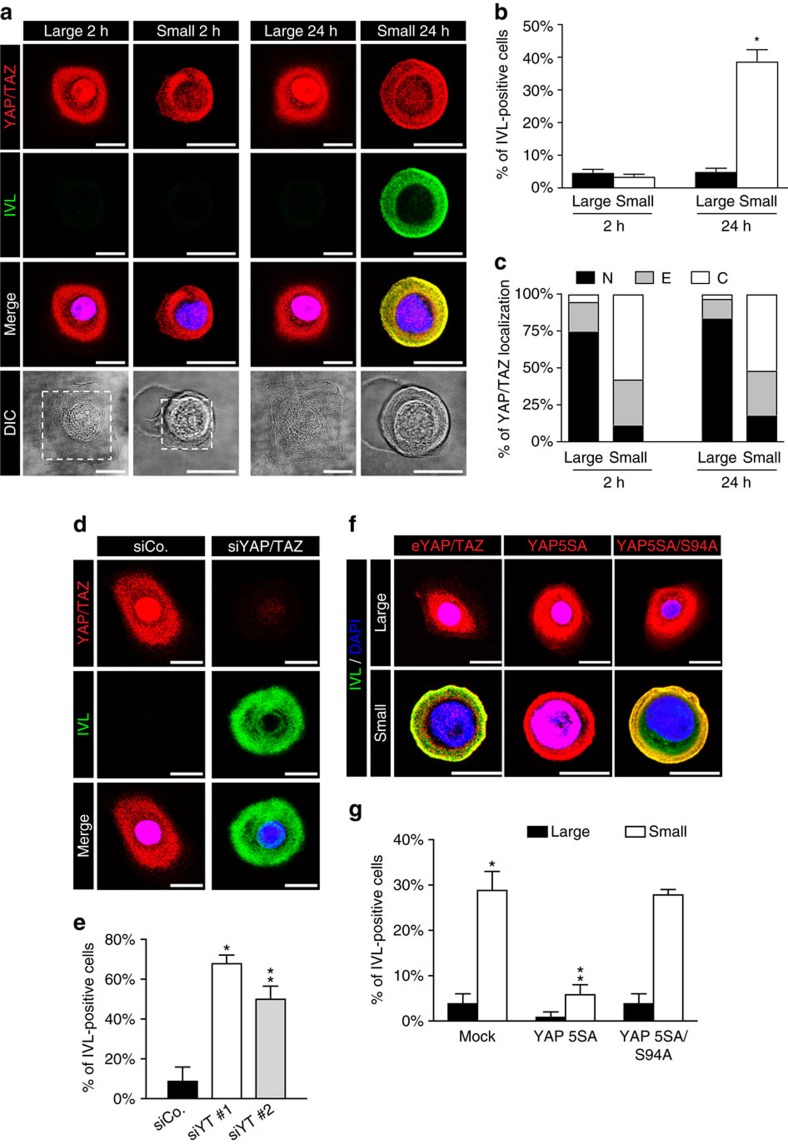
Controlling cell shape regulates the fate of individual epidermal SCs through YAP/TAZ. (**a**) Confocal IF (top) and bright field images (bottom) of neonatal human epidermal keratinocytes (nHEK) plated as individual cells on square microprinted fibronectin islands of 1,024 μm^2^ (large) or 300 μm^2^ (small). Cells were stained for endogenous YAP and TAZ proteins (red) and Involucrin (IVL, green). DAPI (blue) is a nuclear counterstain. Scale bar: 20 μm. Dotted lines highlight microprinted fibronectin islands. (**b**) Quantitation of differentiation of experiments shown in **a**. Bars represent mean+s.e.m. (*P<0.0001, compared to cells on large at 24 h; Student's *t*-test). (**c**) Proportion of nHEK cells displaying preferential nuclear YAP/TAZ localization (N, black), even distribution of YAP/TAZ in nucleus and cytoplasm (E, grey) or cytoplasmic YAP/TAZ (C, white). (**b**,**c**) Data from at least three independent experiments. (**d**) nHEK cells were transfected with control siRNA (siCo.) or with two independent sets of siRNAs against YAP and TAZ mRNAs (siYT #1, siYT #2, see Supplementary Data 2). Staining as in **a**. Scale bar: 20 μm. (**e**) Quantitation of differentiation of nHEK cells treated as in **d**. Bars represent mean+s.d. (*n*=3 independent experiments. *P<0.0001, **P<0.001, compared to siCo.; Student's *t*-test). See also Supplementary Fig. 2a for western blot analysis of nHEK differentiation and Supplementary Fig. 2b for the effect of YAP/TAZ knockdown on keratinocyte proliferation. (**f**) nHEK cells were infected either with an empty vector (Mock) or with the indicated doxycycline-inducible lentiviral YAP constructs. After 24 h, cells were replated on either small or large fibronectin islands, induced with doxycycline for additional 24 h and finally analysed. Confocal images show representative staining for either endogenous YAP/TAZ proteins in Mock-infected cells or the HA-tagged YAP constructs (red), merged with Involucrin (green) and DAPI (blue). Scale bar: 20 μm. See Supplementary Fig. 1d for HA-tag background staining on Mock-infected control cells. (**g**) Quantitation of differentiation of experiments shown in **f**. Bars represent mean+s.d. (*n*=3 independent experiments. *P<0.0001 compared to Mock-infected cells plated on large, **P<0.001 compared to Mock-infected cells plated on small; Student's *t*-test).

We first tested the effect of modulating cell–ECM adhesiveness by comparing the behaviour of individual keratinocytes plated either on small or large microprinted ‘islands' of adhesive fibronectin (Fig. 1a). This manipulation of the physical microenvironment allows controlling the shape of individual cells: after seeding, cells adopted a spread morphology on large islands, and a more rounded, compact shape on small islands. Differentiation was evaluated 2 and 24 h after plating by monitoring the expression of involucrin, a marker of terminal differentiation (Fig. 1b). At 2 h, cells were negative for involucrin on both small and large islands; at 24 h, the number of involucrin-positive cells greatly increased on the small islands, whereas on large islands cells remained protected from differentiation, as previously reported^12^. YAP/TAZ appeared prominently nuclear on large cells but excluded from nuclei in cells on small islands (Fig. 1c).

To investigate the role of YAP/TAZ in shape-induced epidermal SC decisions, we inactivated YAP/TAZ by independent sets of short interfering RNAs (siRNAs) in spread/large cells. YAP/TAZ-depleted cells initiated en-mass differentiation and exited the cell cycle (Fig. 1d,e and Supplementary Fig. 2a,b). These findings correlate with YAP/TAZ nuclear exclusion by differentiating cells adhered to small islands and indicate that YAP/TAZ regulation by cell mechanics is a critical determinant of epidermal SC decisions.

Next, we investigated whether raising YAP/TAZ activity is sufficient to oppose shape-induced differentiation and to preserve epidermal stemness. For this, we infected keratinocytes with lentiviral vectors expressing a doxycycline-inducible version of an activated form of YAP (YAP5SA, lacking inhibitory phosphorylation sites)^21^; as control, we used the YAP5SA carrying the additional S94A mutation, disabling any interaction with TEAD (the main YAP/TAZ-binding platform on DNA)^33^,^34^. Keratinocytes were plated on small fibronectin islands and treated with doxycycline to induce ectopic YAP expression (Fig. 1f). As shown in Fig. 1g, the expression of YAP5SA, but not of YAP5SA/S94A, was sufficient to prevent involucrin expression. Thus a ‘YAP/TAZ ON' biomechanical state impedes cell differentiation, whereas a ‘YAP/TAZ OFF' state promotes it.

The rigidity of the ECM to which cells adhere is a key determinant of the mechanical strains that control cell shape and cytoskeletal organization in living tissues. Such physical forces are essential to regulate proliferation and differentiation in different cell types^35^. In the basal layer of the skin, epidermal stem/progenitor cells are attached to the basement membrane mainly through β1 integrin; loss of this contact stops proliferation and induces differentiation^8^,^32^. Moreover, increases in ECM rigidity and epithelial tension have been proposed to coordinate re-epithelialization during skin wound healing^10^.

**Figure 2:**
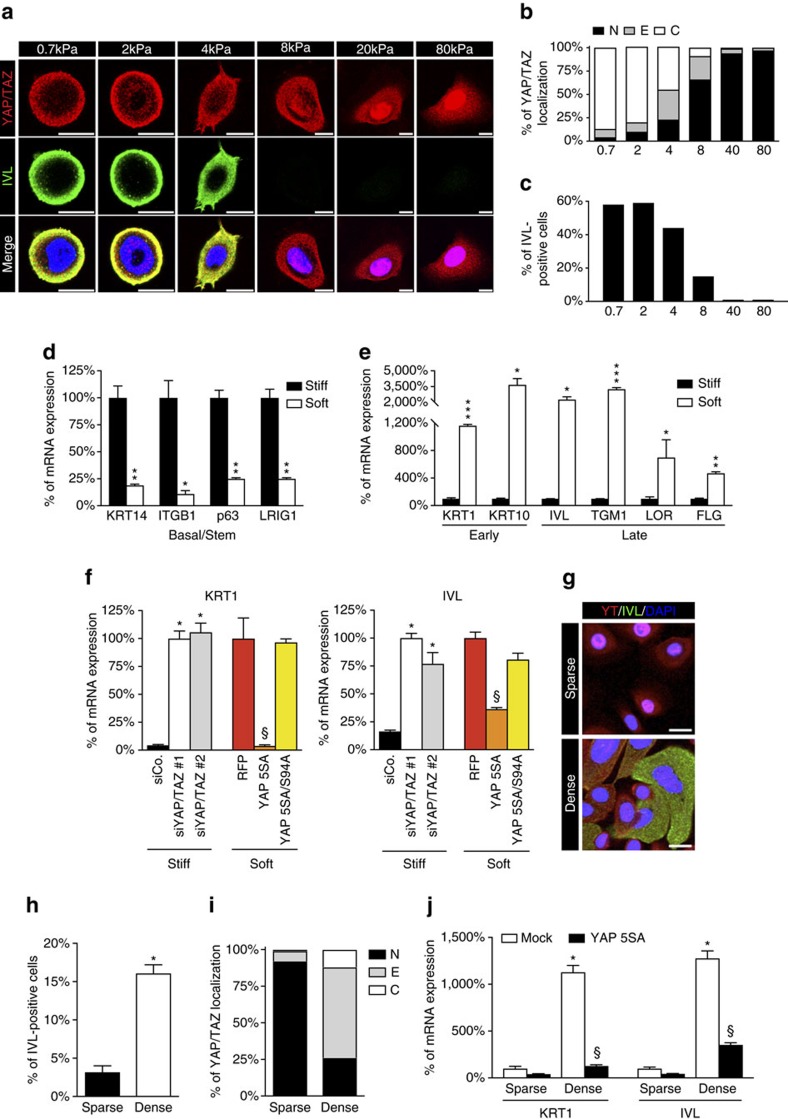
Soft ECM or high density lead to epidermal SC differentiation through YAP/TAZ inhibition. (**a**) Confocal IF images of YAP/TAZ (red) and Involucrin (green) proteins in nHEK cells plated for 24 h on a series of fibronectin-coated polyacrylamide hydrogels ranging from 0.7 to 80 kPa elastic modulus. DAPI (blue) is a nuclear counterstain. Scale bar: 20 μm. (**b**,**c**) The analysis of YAP/TAZ subcellular distribution (**b**) and the quantitation of differentiation (**c**) induced by ECM substrates with different elasticity. Data from one representative experiment out of three are shown. (**d**,**e**) Soft ECM substrates induce upregulation of keratinocyte differentiation genes, while it downregulates basal/stem markers. Relative mRNA expression values were normalized to the stiff condition for each gene analysed. Bars represent mean+s.d. *P<0.05, **P<0.01, ***P<0.001 compared to siCo.; Student's *t*-test). See Methods section for reproducibility of experiments. (**f**) Quantitation of differentiation of nHEK cells transfected with the indicated siRNAs or infected with the indicated lentiviral constructs (as in Fig. 1d,f, respectively), plated on stiff and soft conditions and assayed by qRT–PCR for *KRT1* and *IVL*. Data were normalized to the siYAP/TAZ-transfected cells on stiff (white bar, left) or to the RFP-infected cells on soft (red bar, right). Bars represent mean+s.d. (*P<0.001 compared to siCo., ^§^*P*<0.001 compared to RFP; one-way analysis of variance). See Methods section for reproducibility of experiments. (**g**) Confocal images of nHEK cells, stained as in **a**, plated as sparse or dense cultures. Scale bar: 20 μm. (**h**) Quantitation of differentiation of nHEK shown in **g**. Bars represent mean+s.d. (*n*=3 independent experiments. *P<0.0001; Student's *t*-test). (**i**) YAP/TAZ nucleo/cytoplasmic localization was scored as previously described (*n*=3 independent experiments). (**j**) nHEK cells infected with the indicated doxycycline-inducible lentiviral constructs were plated to obtain sparse or dense cultures. After 48 h, cells were analyzed by qRT–PCR for keratinocyte differentiation. Data were normalized to the Mock-infected cells in sparse condition. Bars represent mean+s.d. (*P<0.0001 compared to sparse mock, ^§^*P*<0.0001 compared to dense mock; one-way analysis of variance). See Methods section for reproducibility of experiments.

To study whether the fate of human epidermal SCs is also affected by YAP/TAZ mechanotransduction regulated by ECM elasticity, we first prepared a series of fibronectin-coated polyacrylamide hydrogels with different elastic modulus, ranging from 0.7 to 80 kPa, as validated by atomic force microscopy (Fig. 2a). YAP/TAZ were cytoplasmic on 0.7 and 2 kPa, evenly distributed between the cytoplasm and nucleus at 4 kPa and shifted to a predominantly nuclear localization in cells seeded on hydrogels at higher rigidities (8–80 kPa) (Fig. 2b). Involucrin expression paralleled YAP/TAZ nuclear exclusion: as ECM rigidity increased, the proportion of cells that underwent terminal differentiation progressively decreased (Fig. 2c). Mechano-dependent control of epidermal stemness and differentiation was further confirmed by quantitative reverse transcriptase polymerase chain reaction (qRT–PCR; Fig. 2d,e). When compared to cells seeded on stiff substrates (80 kPa hydrogels or fibronectin-coated glass), cells seeded on a soft substrate (1 kPa hydrogels) turned on robust expression of a panel of terminal differentiation markers—such as Loricrin, Filaggrin, Keratin 1, Keratin 10, Transglutaminase1—(Fig. 2e) and concomitantly lost the expression of basal and SC markers (Keratin 14, β1 integrin, p63 and LRIG1; Fig. 2d) and of the proliferation marker Ki67 (Supplementary Fig. 2c). Inactivation of YAP/TAZ with independent siRNAs induced differentiation of cells plated on rigid substrates, phenocopying the effect of ECM softness (Fig. 2f and Supplementary Fig. 2d,e). Oppositely, raising YAP/TAZ activity by transduction of a doxy-inducible lentiviral vector expressing YAP prevented differentiation of cells plated on soft substrates (Fig. 2f and Supplementary Fig. 2f).

High cell density in postconfluent epithelial monolayers is yet another condition known to turn-off YAP/TAZ activity through attenuation of cellular mechanotransduction^36^. Indeed, as any further spreading of a cell monolayer is limited by spatial constraints, crowding imposes cells to be attached to a progressively smaller adhesive ECM area until a critical threshold is reached, below which YAP/TAZ are completely inactivated^21^. Of note, we found that, in analogy with the mechanobiology assay above described, postconfluent cell density also triggered YAP/TAZ nuclear exit and differentiation (Fig. 2g–i), in a manner opposed by YAP overexpression (Fig. 2j). The collective set of data presented so far indicates YAP/TAZ as unifying biomechanical sensors integrating distinct aspects of the physicality of the cell's environment and, as such, controlling epidermal SC decisions.

**Figure 3:**
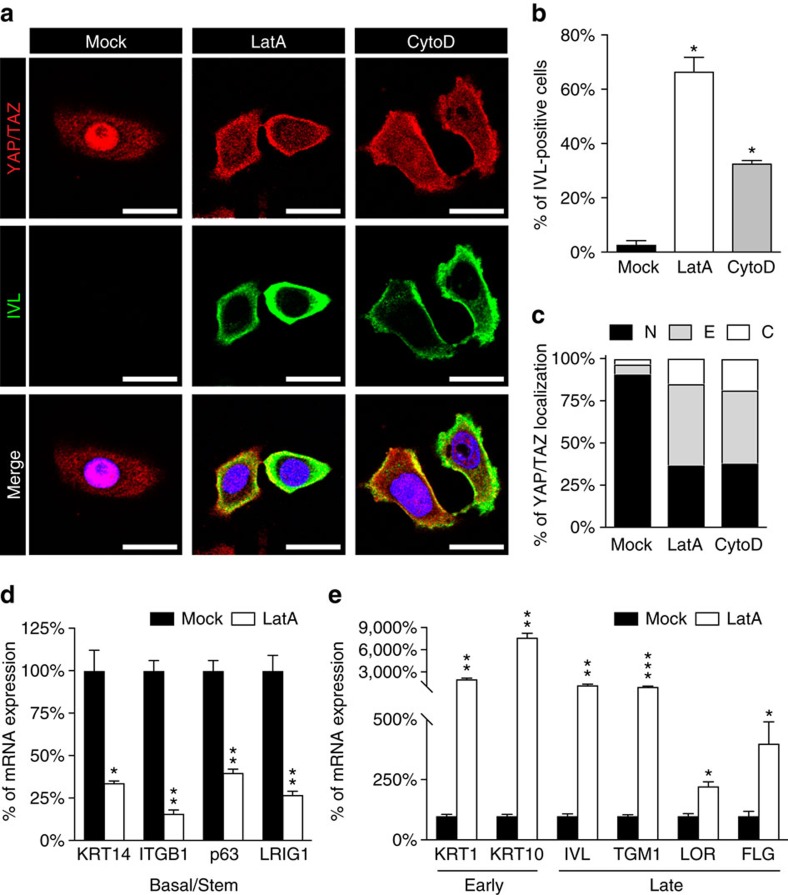
F-actin preserves epidermal stemness by sustaining YAP/TAZ and opposing differentiation. (**a**) IF microscopic images of nHEK cells treated for 24 h with vehicle (Mock), 0.8 μM latrunculin A (LatA) or 2.5 μM cytochalasin D (CytoD). Images show the endogenous YAP/TAZ proteins (red) and the Involucrin differentiation marker (IVL, green). DAPI (blue) is a nuclear counterstain. Scale bar: 20 μm. (**b**) Quantitation of Involucrin expression after treatment for 24 h with actin inhibiting drugs as in **a**. Bars represent mean+s.d. (*n*=3 independent experiments. *P<0.0001 compared to Mock-treated cells; Student's *t*-test). (**c**) Proportion of YAP/TAZ subcellular distribution in nHEK cells treated with the indicated drugs and analysed as previously described. Bars represent mean of three independent experiments. (**d**,**e**) nHEK cells treated for 24 h with vehicle (Mock) or 0.8 μM latrunculin A (LatA) were analysed by qRT–PCR for the expression of either basal and stem marker genes (*KRT14*, *ITGB1*, *p63*, *LRIG1*) (**d**) or early (*KRT1*, *KRT10*) and late (*IVL*, *TGM1*, *LOR*, *FLG*) differentiation genes (**e**). Notably, LatA potently promoted differentiation as shown by the upregulation of the indicated markers. At the same time, disrupting F-actin integrity is sufficient to reduce the expression of basal (*KRT14*) and SC (*ITGB1*, *p63* and *LRIG1*) markers. Bars represent mean+s.d. *P<0.05, **P<0.01, ***P<0.001 compared to Mock; Student's *t*-test). See Methods section for reproducibility of experiments. See also Supplementary Fig. 2g for the effect of F-actin-inhibiting drugs on YAP/TAZ transcriptional activity in keratinocytes.

Cells are active mechanical entities and respond to the physical inputs received from their environment by modulating actomyosin tension and by restructuring the architecture of their F-actin cytoskeleton. It has been previously shown that epidermal SCs forced to adhere on small adhesive areas organize a thick layer of cortical F-actin, leading to an increase in total F-actin levels^12^. This measurement has prompted other authors to propose that the ratio between F-actin versus free G-actin serves as determinant of epidermal cell fate decisions informed by cell mechanics, with high F-actin inducing differentiation^12^. At difference with this notion, we found that treatment of epidermal SCs with F-actin inhibitors, such as latrunculin A (that sequesters G-actin) or cytochalasin D (blocking F-actin polymerization) potently promoted keratinocyte differentiation on rigid and large adhesive areas (Fig. 3a,b,d,e). Consistently, F-actin inactivation caused quantitative inhibition of YAP/TAZ nuclear localization (Fig. 3c) and transcriptional activities (Supplementary Fig. 2g). These results are consistent with the previously reported positive role of F-actin as intracellular transducer of mechanical stimuli^21^,^22^, ultimately resulting in YAP/TAZ activation, and suggest that a YAP/TAZ-proficient F-actin organization is a main inhibitor of epidermal differentiation.

### YAP/TAZ-mechanotransduction inhibits Notch in epidermal SCs

Next, we aimed to determine what signals operate downstream of YAP/TAZ in order to regulate epidermal differentiation. Notch signalling is a fundamental negative regulator of epidermal stemness and an inducer of terminal differentiation^25^,^27^,^37^. The function of Notch in epidermal cells appeared thus antithetic to those of YAP/TAZ and overlapping to those triggered by low mechanical stimuli. These analogies suggested the intriguing possibility that biomechanical regulation of YAP/TAZ could reflect into biomechanical regulation of Notch signalling.

**Figure 4:**
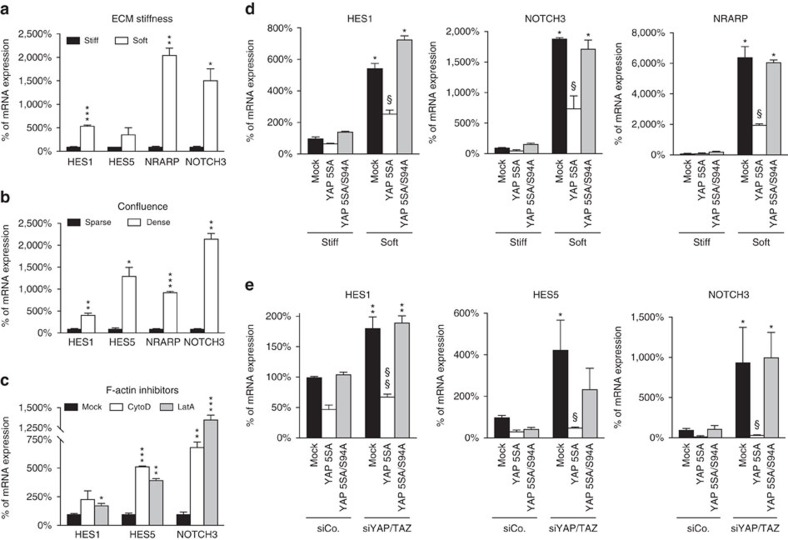
YAP/TAZ regulate epidermal SC differentiation through Notch inhibition. (**a**,**b**) Mechanical forces regulate Notch signalling. nHEK cells were plated either on fibronectin-coated plastic dishes (stiff) and fibronectin-coated 1 kPa polyacrylamide hydrogels (soft) (**a**) or at sparse and dense culture conditions (**b**). After 24 (**a**) or 48 h (**b**), cells were harvested and analysed by qRT–PCR for the expression of the Notch target genes *HES1*, *HES5*, *NRARP* and *NOTCH3*. For each gene, data were normalized respectively to the stiff (**a**) or the sparse (**b**) condition (black bars). (**c**) F-actin-targeting drugs activate Notch signalling. nHEK cells treated for 24 h with control vehicle (Mock), 0.8 μM latrunculin A (LatA) or 2.5 μM cytochalasin D (CytoD) were analysed by qRT–PCR for the expression of the Notch target genes *HES1*, *HES5* and *NOTCH3*. (**a**–**c**) Bars represent mean+s.d. (*P<0.05, **P<0.01, ***P<0.001 compared to the respective stiff (**a**), sparse (**b**) or Mock (**c**) controls; Student's *t*-test). (**d**,**e**) YAP/TAZ activity regulates Notch signalling. (**d**) nHEK cells infected with the empty vector (Mock) or with the indicated siRNA-insensitive doxycycline-inducible lentiviral YAP constructs were plated either on stiff or soft ECM substrates, as in Fig. 2f. Data were normalized to the Mock-infected cells plated on stiff. Bars represent mean+s.d. (*P<0.0001 compared to the Mock-infected cells on stiff, ^§^*P*<0.0001 compared to the Mock-infected cells on soft; two-way analysis of variance (ANOVA)). (**e**) nHEK cells infected as in **d** were transfected with control (siCo.) or YAP/TAZ siRNAs (siYAP/TAZ), and 8 h posttransfection, cells were treated with doxycycline for 40 h. Cells were analysed by qRT–PCR for the induction of Notch target genes. Data were normalized to the Mock-infected cells transfected with siCo. Bars represent mean+s.d. (*P<0.001, **P<0.0001 compared to Mock-siCo; ^§^*P*<0.001, ^§§^*P*<0.0001 compared to the Mock-siYAP/TAZ; two-way ANOVA). (**a**–**e**) See Methods section for reproducibility of experiments.

To address this hypothesis, we first tested whether epidermal SCs exposed to low mechanical inputs indeed turned on Notch signalling. As shown in Fig. 4a cells plated on soft ECM upregulated the Notch target genes *HES1*, *HES5*, *NRARP* and *NOTCH3*. Similar results were obtained in cells cultured at high density (Fig. 4b) or after inhibition of F-actin polymerization (Fig. 4c). To address more directly whether mechanical control of Notch signalling is in fact a consequence of mechanical regulation of YAP/TAZ, we asked whether raising YAP levels could intercept the activation of Notch signalling mediated by a compliant ECM. For this, cells were transduced with lentiviral vectors encoding for YAP5SA or controls (mock/empty vector and YAP5SA/S94A), placed on soft hydrogels (1 kPa) and analysed by qRT–PCR for the expression of Notch target genes. As shown in Fig. 4d, induction of the Notch targets *HES1*, *NRARP* and *NOTCH3* triggered by a soft ECM was significantly blunted by the expression of YAP5SA.

To further validate the notion that YAP/TAZ oppose Notch activity in epidermal SCs, we tested whether YAP/TAZ inactivation also triggers Notch activation, phenocopying low mechanical strains. We found that silencing YAP/TAZ in mechanically stiff conditions caused an upregulation of Notch target genes (Fig. 4e) and enhanced the levels of the transcriptionally active fragment of the Notch1 receptor (N1ICD) (Supplementary Fig. 3a). Suppression of Notch signalling relied on YAP/TAZ-dependent transcription, as the effect of YAP/TAZ inhibition by knockdown or soft ECM was rescued by a siRNA-insensitive YAP5SA but not by the transcriptionally deficient YAP mutant YAP5SA/S94A (Fig. 4d,e).

**Figure 5:**
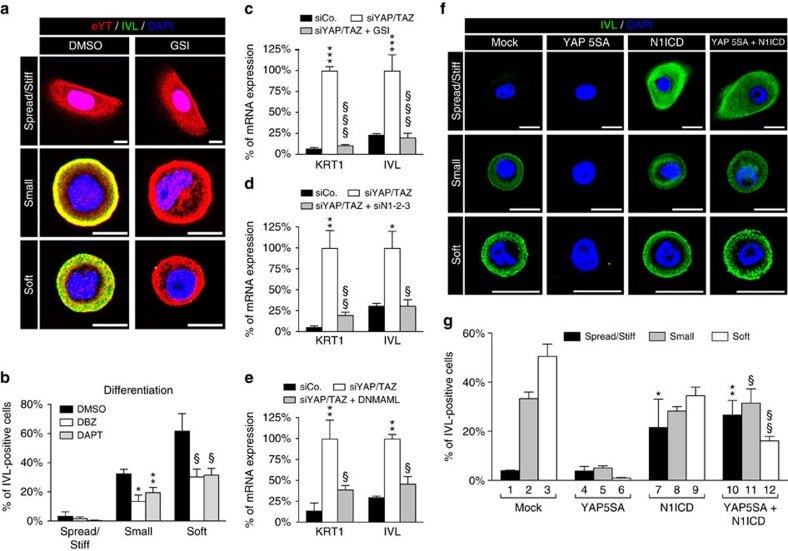
Notch is epistatic to YAP/TAZ and required for mechano-induced epidermal SC differentiation. (**a**,**b**) nHEK cells were seeded for 24 h on fibronectin-coated coverslips (spread/stiff), fibronectin islands of 300 μm^2^ (small) or fibronectin-coated hydrogels of 1 kPa (soft) and concomitantly treated with control vehicle (DMSO) or with two independent γ-secretase inhibitors (GSIs; DAPT, 20 μM; DBZ, 2.5 μM). (**a**) Confocal images of cells plated as described and stained for endogenous YAP/TAZ (eYT, red), Involucrin (IVL, green) and nuclear compartment (DAPI, blue). Representative images of cells treated with control vehicle (DMSO) or DBZ (GSI) are shown. Scale bar: 20 μm. (**b**) Quantitation of differentiation of data shown in **a** (as in Figs 1 and 2). Bars represent mean+s.d. (*n*=3 independent experiments. *P=0.009 and **P=0.0003 compared to DMSO-treated cells on small, ^§^*P*<0.0001 compared to DMSO-treated cells on soft; two-way analysis of variance (ANOVA)). (**c**–**e**) nHEK cells transfected with control (siCo.) or YAP/TAZ siRNAs (siYAP/TAZ) were either treated with DBZ GSI (**c**), cotransfected with a combination of siRNAs targeting Notch1, Notch2 and Notch3 receptors (siN1-2-3) (**d**) or infected with a doxycycline-inducible lentiviral vector encoding the dominant-negative form of MAML1 (DNMAML) (**e**). After 48 h from siRNA transfection, cells were analysed by qRT–PCR for *KRT1* and *IVL*. Data were normalized to the control-treated siYAP/TAZ-transfected cells (white bar). Bars represent mean+s.d. (*P<0.01, **P<0.001, ***P<0.0001 compared to siCo; ^§^*P*<0.01, ^§§^*P*<0.001, ^§§§^*P*<0.0001 compared to siYAP/TAZ; two-way ANOVA). See Methods section for reproducibility of experiments. (**f**) Confocal images showing representative staining for IVL of nHEK cells infected with the indicated constructs and plated on spread/stiff, small or soft conditions. Scale bar: 20 μm. (**g**) Quantitation of differentiation of nHEK cells treated and stained as in panel (**f**). Only infected cells were scored for the presence of the Involucrin staining. Bars represent mean+s.d. (*n*=3 independent experiments. *P≤0.005 lane 7 versus lane 1/4, **P≤0.0005 lane 10 versus lane 1/4, ^§^*P*<0.0001 lane 11 versus lane 5, ^§§^*P*=0.02 lane 12 versus lane 6; two-way ANOVA).

Next, we tested whether Notch signalling is required for mechano-dependent differentiation by treating cells with γ-secretase inhibitors. Epidermal SCs seeded on small adhesive areas (300 μm^2^) or on a soft substrate (1 kPa) were treated either with vehicle (DMSO) or with two structural unrelated γ-secretase inhibitors *N*-[*N*-(3,5-difluorophenacetyl)-l-alanyl]-S-phenylglycine t-butyl ester (DAPT) or Dibenzazepine (DBZ) and their differentiation measured by involucrin expression, by IF (Fig. 5a,b) and qRT–PCR (Supplementary Fig. 3b,c). As shown in Fig. 5b, shape-dependent differentiation was diminished by DAPT or DBZ treatment. Similar results were obtained with cells plated on a soft substrate (Fig. 5b and Supplementary Fig. 3b). Collectively, the results indicate that Notch signalling is a mechanically regulated effector of epidermal cell fate decisions.

If Notch activation is downstream of YAP/TAZ inhibition for epidermal SC differentiation, then inhibition of Notch should rescue the effects of YAP/TAZ knockdown. For this type of epistatic experiments, three different strategies were employed to oppose Notch signalling: (i) inhibition of γ-secretase activity; (ii) combined knockdown of Notch receptors by siRNA transfection; and (iii) overexpression of the dominant-negative form of MAML1 (DN-MAML1) inhibiting the transcriptional effects of Notch at the chromatin level^38^. All these redundant experimental approaches provided consistent results, confirming that cell differentiation of YAP/TAZ-depleted cells was prevented by Notch inhibition, as revealed by analyses of early (KRT1) and late (IVL) differentiation markers (Fig. 5c–e, see also additional controls in Supplementary Fig. 3d–g).

To further validate this pathway, we then tested whether the sole activation of the Notch pathway was sufficient to promote differentiation in cells with elevated YAP/TAZ levels. We found that primary keratinocytes overexpressing the N1ICD fragment readily differentiated even when cultured on a stiff substrate (Fig. 5f,g, compare lanes 1/4 with lanes 7 and 10; and Supplementary Fig. 3h). Overexpression of N1ICD was essentially inconsequential on the differentiation of cells plated on small or soft substrates, as expected by the fact that these cells already display low YAP/TAZ and sufficient activation of endogenous Notch signalling; yet, N1ICD overexpression restored differentiation in YAP-expressing cells cultured under the same low-tension microenvironments (Fig. 5f,g, compare lanes 5/6 with 11/12).

Taken together, these data indicate that Notch signalling is downstream of YAP/TAZ and that YAP/TAZ mechanical activation preserves the undifferentiated state of human epidermal SCs through inhibition of Notch.

### *In vivo* validation of the YAP/TAZ and Notch connection

**Figure 6:**
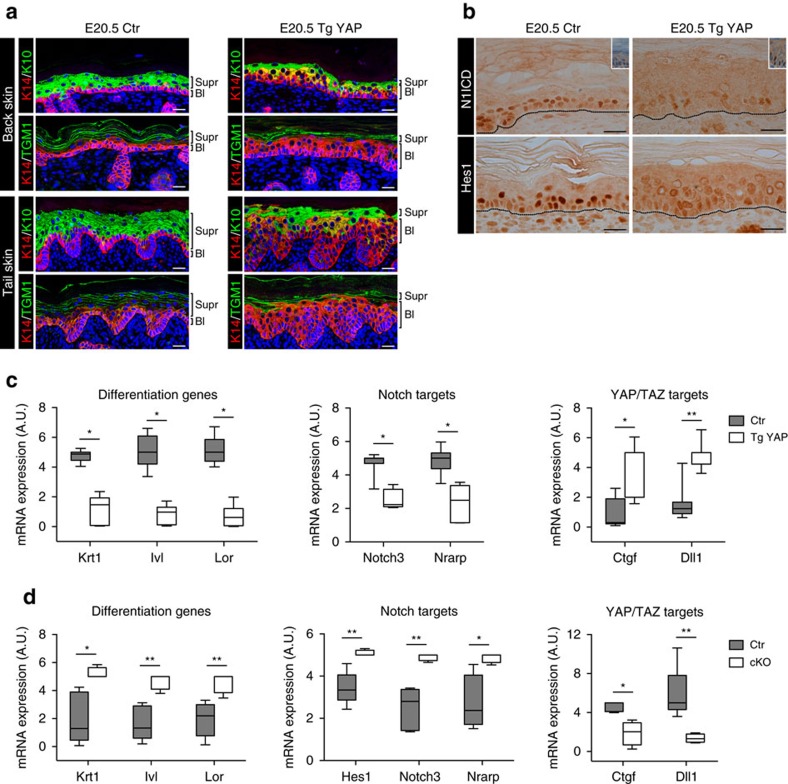
*In vivo>* validation of YAP/TAZ as inhibitors of Notch signalling in epidermis. (**a**–**c**) YAP induction inhibits epidermal differentiation and Notch signalling *in vivo*. (**a**) YAP overexpression in YAP-transgenic (Tg YAP) mice leads to the expansion of the basal layer and diminished terminal differentiation. IF on the back and tail skin of E20.5 embryos reveals increased thickness of the KRT14-positive basal layers (Bl) with concomitant reduction of the differentiated KRT10- and TGM1-positive suprabasal layers (Supr) compared to control littermates (Ctr). K14, keratin 14; K10, keratin 10. Scale bar: 20 μm. See also Supplementary Fig. 4a for additional data at E18.5. (**b**) YAP inhibits Notch signalling in epidermis. Immunohistochemistry on the back skin of Tg YAP at E20.5 embryos shows decreased Notch signalling in the epidermis compared to control littermates (Ctr), as visualized by the greatly reduced levels of the N1ICD fragment (top panel) and Hes1 (bottom panel). The insets (0.5 × ) show immunohistochemistry for Yap and nuclear counterstaining in Ctr and Tg YAP mice, as control of YAP overexpression. Scale bar: 20 μm. (**c**) qRT–PCR on skin biopsies of mice as in **a**,**b**. YAP overexpression inhibits the expression of both terminal differentiation markers of epidermis (*Krt1*, *Ivl*, *Lor*) and Notch transcriptional targets (*Notch3*, *Nrarp*), while it upregulates the YAP/TAZ target genes (*Ctgf*, *Dll1*). Relative mRNA expression values were normalized to an internal sample for each experiment and represented in arbitrary units (A.U.) as box plots (*n*=8 for Ctr and *n*=7 for Tg YAP, from three independent experiments. *P<0.0001, **P<0.01 compared to Ctr; Student's *t*-test). (**d**) YAP/TAZ double conditional knockout (cKO) induces terminal differentiation and Notch signalling *in vivo*. qRT–PCR analysis on the epidermis from E17.5 embryos shows an upregulation of both the terminal differentiation markers (*Krt1*, *Ivl*, *Lor*) and Notch transcriptional targets (*Hes1*, *Notch3*, *Nrarp*) in Yap/Taz cKO mice compared to controls (Ctr). The reduced YAP/TAZ transcriptional activity in cKO embryos is confirmed by the dowregulation of the target genes *Ctgf* and *Dll1*. Relative mRNA expression values were normalized to an internal sample for each experiment and represented in A.U. as box plots (*n*=5 for Ctr and *n*=4 for cKO, from three independent experiments. *P<0.05, **P<0.01 compared to Ctr; Student's *t*-test). Box plots in **c**,**d** show the median, interquartile range, minimum and maximum values.

Next, we aimed to validate the YAP/TAZ and Notch connection at the genetic level, using transgenic mice. For this, we first tested the effect of YAP overexpression in *K5-rtTA; tetO-YAP*^*S127A*^ allowing doxycyline-inducible expression of YAPS127A in the basal layer of the skin. Doxycyline was administered in the drinking water of pregnant females at E14.5 or E16.5 days postcoitum, and the consequences of YAP expression were evaluated in the tail and back skin of the progeny after 96 h. In line with previous reports^19^,^20^, YAP triggered massive expansion of the KRT14-positive basal layer; this occurred at the expense of differentiated KRT10- and TGM1-positive suprabasal layers (Fig. 6a and Supplementary Fig. 4a). Consistently with our model, expansion of the epidermal SC compartment was accompanied by decreased Notch signalling, as visualized through immunohistochemistry by the greatly reduced levels of the N1ICD fragment and Hes1 (Fig. 6b). The *in vivo* consequences of YAP activation on Notch signalling was also confirmed by qRT–PCR on skin biopsies, showing attenuated expression of Notch transcriptional targets (*Notch3*, *Nrarp*) and reduced expression of differentiation markers (*Krt1*, *Ivl*, *Lor*) (Fig. 6c).

To address the requirement of YAP/TAZ as inhibitors of Notch signalling, we exploited *K14-CreER; YAP*^*fl/fl*^*; TAZ*^*fl/fl*^ mice^33^. Tamoxifen was injected intraperitoneally in pregnant females to activate CRE in the basal layer of E13.5 embryos, and the progeny was analyzed after 96 h by qRT–PCR (Fig. 6d). The data reveal upregulation of Notch target genes (*Hes1*, *Notch3*, *Nrarp*) and concomitant induction of skin differentiation markers (*Krt1*, *Ivl*, *Lor*) in YAP/TAZ conditional knockout samples. Collectively, these results validate *in vivo* the antithetic relationship between YAP/TAZ activity and Notch in epidermal progenitors, as previously deduced *in vitro* from human epidermal SCs.

### YAP/TAZ transcriptionally control Notch regulators

**Figure 7:**
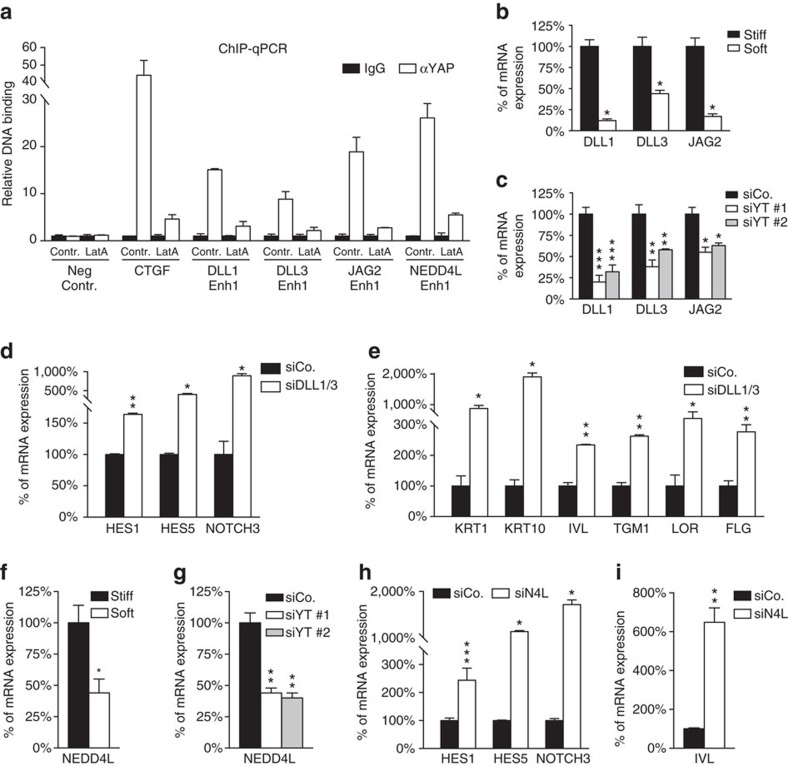
YAP/TAZ transcriptionally control Notch regulators. (**a**) Validation by ChIP-qPCR of YAP/TAZ-binding sites identified through combination of YAP/TAZ ChIP-seq and Hi-C (see Supplementary Data 1). ChIP-qPCR of the *CTGF* promoter is a positive control; *HBB* is a negative control locus (Neg Contr.). Data from two biological replicates (mean+s.d.) from one representative experiment out of three are shown. (**b**,**c**) Mechano-YAP/TAZ-dependent regulation of Notch ligands. nHEK cells were either plated on stiff or soft conditions (**b**) or transfected with control siRNA (siCo.) or with independent siYAP/TAZ (**c**). Cells were analysed by qRT–PCR for *DLL1*, *DLL3* and *JAG2*. Values were normalized and statistically compared to stiff (**b**) or to siCo. treated (**c**). Bars represent mean+s.d. (*n*=3 independent experiments. *P<0.0001 in **b**; ***P<0.0001, **P<0.01, *P<0.02 in **c**; two-way analysis of variance (ANOVA)). (**d**,**e**) nHEK cells transfected with control siRNA (siCo.) or with a mix of siRNAs targeting both DLL1 and DLL3 mRNAs (siDLL1/3) were replated at single-cell density to minimize the effect of *trans*-signalling of Notch ligands. qRT–PCR for the expression of the Notch target genes *HES1*, *HES5* and *NOTCH3* (**d**) and for a panel of differentiation markers (*KRT1*, *KRT10*, *IVL*, *TGM1*, *LOR*, *FLG*) (**e**). Bars represent mean+s.d. (*P<0.0001, **P<0.05, compared to siCo.; two-way ANOVA). (**f**,**g**) Mechano-YAP/TAZ-dependent regulation of NEDD4L. nHEK cells treated as previously described (**b**,**c**) were analysed by qRT–PCR for the expression of *NEDD4L* gene. Values were normalized, respectively, to the stiff condition (**f**) or to the siCo.-treated cells (**g**). Bars represent mean+s.d. (*n*=3 independent experiments. *P=0.018 compared to stiff, **P<0.001 compared to siCo.; one-way ANOVA). (**h**,**i**) nHEK cells transfected with control siRNA (siCo.) or with a NEDD4L-targeting siRNA were analysed by qRT–PCR for the expression of the Notch target genes *HES1*, *HES5* and *NOTCH3* (**h**) and for the *IVL* differentiation marker (**i**). Bars represent mean+s.d. (*P<0.0001, **P<0.01, ***P<0.05, compared to siCo.; two-way ANOVA). See Methods section for reproducibility of experiments.

Data presented so far indicate that mechanical inhibition of Notch by YAP/TAZ requires YAP/TAZ-dependent transcription, prompting us to hypothesize that, being transcriptional coactivators, YAP/TAZ may affect the Notch cascade by directly controlling the transcription of Notch inhibitors. Surprisingly, little is known on how Notch signalling components are regulated at the transcriptional level. YAP/TAZ control transcription mainly from enhancers located very distantly from the transcription start site (TSS) of the gene they regulate, to which YAP/TAZ-bound enhancers physically interact by chromatin looping^33^. We thus decided to entertain our hypothesis by first carrying out a bioinformatic search of candidate YAP/TAZ direct targets related to Notch signalling. To this end, we combined YAP/TAZ ChIP-Seq and high-resolution chromatin conformation capture data (Hi-C) to generate a virtual YAP/TAZ chromatin ‘interactome map', listing YAP/TAZ-bound *cis*-regulatory elements and the genes that they regulate (see Supplementary Data 1 and Supplementary Methods). From this list, our attention was captured by the fact that several Notch ligands, such as *DLL1*, *DLL3* and *JAG2*^26^, are all associated with several YAP/TAZ-bound enhancers. Chromatin immunoprecipitation quantitative polymerase chain reaction (ChIP-qPCR) experiments confirmed that YAP/TAZ were specifically bound to these chromatin regions in epidermal progenitors cultured on stiff substrates but not in cells in which the F-actin cytoskeleton has been disrupted (Fig. 7a and Supplementary Fig. 5a). Consistently, by qRT–PCR, the expression of *DLL1*, *DLL3* and *JAG2* was mechano-dependent, being downregulated both in cells plated on soft ECM (Fig. 7b) or treated with F-actin inhibitors (Supplementary Fig. 5c) or upon YAP/TAZ knockdown (Fig. 7c). Thus these Delta-like ligands represent direct YAP/TAZ targets, as also validated for *Dll1* by YAP gain- and loss-of-function in skin biopsies of *K5-rtTA; tetO-YAP*^*S127A*^ and *K14-CreER; YAP*^*fl/fl*^*; TAZ*^*fl/fl*^ mice (Fig. 6c,d). The ability of YAP/TAZ to turn ON Delta-like ligands is intriguing, as these ligands are mainly known to stimulate Notch activity in neighbouring cells (that is, non-cell autonomously, also called ‘in *trans*' signalling), but, at the same time, are also relevant to lower the baseline of ligand-independent Notch activation when expressed cell-autonomously (that is, ‘in *cis*')^28^,^39^. Indeed, although Notch signalling is a potent inducer of differentiation in the suprabasal layers of the skin, basal expression of Notch ligands in fact protect basal progenitors from differentiation^9^; not by chance DLL1 is considered the main marker of epidermal SCs^29^, and DLL3 is known to have only Notch-inhibitory functions^40^. It is worth nothing that, consistently with the cell-autonomous-inhibitory role of DLL ligands, YAP/TAZ functioning as Notch inhibitor does not require cell–cell interactions, as here we found that epidermal SC differentiation driven by mechanical stimuli occurs irrespectively of cell–cell contact (that is, as in single cells seeded on small ECM islands or as sparse cultures on soft hydrogels) (see Figs 1 and 2).

Functionally, we found that dual inactivation of DLL1 and DLL3 was sufficient to upregulate Notch signalling in epidermal progenitors, as visualized by induction of the direct Notch targets *HES1*, *HES5* and *NOTCH3* mRNAs (Fig. 7d). Notably, raised levels of Notch signalling upon DLL1/DLL3 knockdown were sufficient to initiate cell differentiation, as determined by activation of multiple differentiation markers (Fig. 7e).

Another YAP/TAZ direct target—first identified bioinformatically and then experimentally validated (Fig. 7a and Supplementary Fig. 5b)—is NEDD4L, a member of the HECT ubiquitin-ligase family^41^. NEDD4 family members have been shown to oppose Notch-dependent differentiation in both mammalian and *Drosophila* cells by suppressing ligand-independent activation of Notch^30^,^42^,^43^,^44^,^45^. NEDD4L was downregulated in cells plated on soft ECM (Fig. 7f), treated with F-actin inhibitors (Supplementary Fig. 5d) or upon YAP/TAZ knockdown (Fig. 7g). Knockdown of the sole NEDD4L also promotes Notch activation and differentiation (Fig. 7h,i). Collectively, these results indicate that YAP/TAZ sets in motion partially overlapping systems that are required to protect epidermal SCs from cell-autonomous self-activation of Notch signalling.

## Discussion

**Figure 8:**
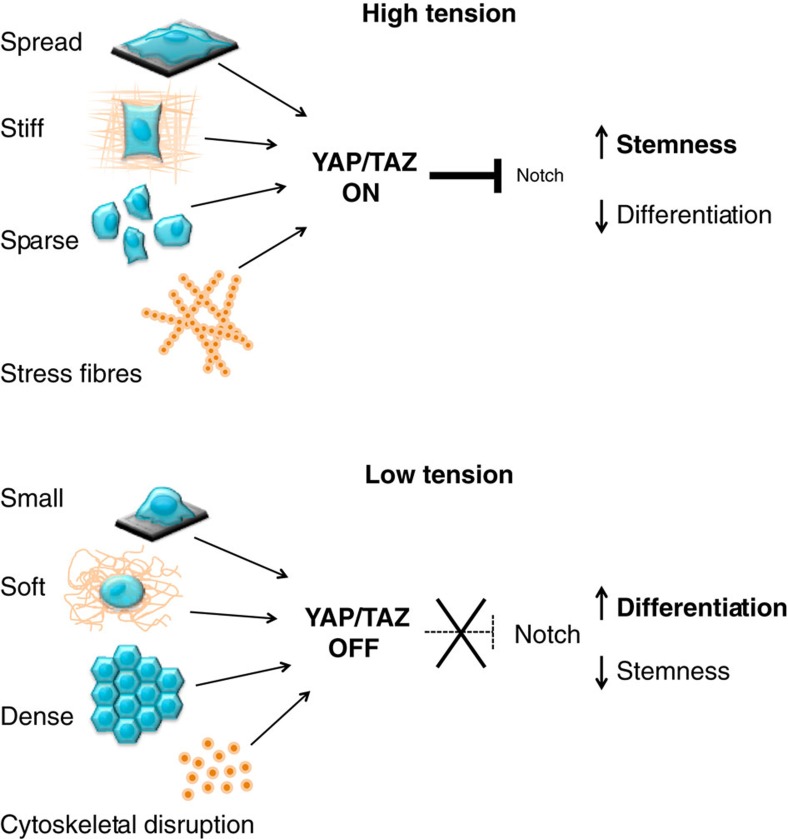
Cell mechanics drives differentiation and stemness in epidermal SCs through YAP/TAZ. See Discussion section for details.

The findings here presented identify a mechanism by which the physical properties of the cell microenvironment can be transmitted to epithelial SCs to regulate a critical decision: remain undifferentiated to expand the SC pool or terminally differentiate. More specifically, we first characterized the action of YAP/TAZ as mediators of mechanical signalling in human epidermal progenitors: YAP/TAZ activation preserves the undifferentiated state downstream of different biomechanical signals, such as cell stretching, adhesion to a rigid extracellular matrix or low cell density. Oppositely, YAP/TAZ inactivation in cells experiencing low mechanical signalling is instrumental for loss of stemness and differentiation (Fig. 8). Thus the demonstration that YAP/TAZ-mechanotransduction can orient the behaviour of normal epithelial stem/progenitors downstream of geometrical and physical signals is a relevant novelty of this work. We offer a unifying model for mechano-responsiveness taking into account previous reported mechanisms for shape-, softness- and high cell-density-induced epidermal SC differentiation^12^,^13^,^14^. Elevated F-actin/G-actin ratios have been connected to shape-induced differentiation via activation of the transcription factor SRF^12^. However, we found that conditions known to reduce F-actin levels, such as soft ECM^5^,^46^ and LatA treatment, indeed induced differentiation, even in the presence of SRF inhibition (Supplementary Fig. 2h). It remains possible that YAP/TAZ and SRF may exert their effects in basal and differentiated cells, respectively, perhaps reflecting differentiation-specific cytoskeletal organizations. Alternatively, as SRF has been shown to induce transcription of actin and actin regulators^47^, SRF may contribute to mechanotransduction by sustaining YAP function. Further work is required to dissect these interesting possibilities. A soft ECM has been also proposed to signal through extracellular signal–regulated kinase/mitogen-activated protein kinase but with unclear nuclear end points^14^. Here we found that shape, ECM rigidity and high density all converge to regulate epidermal cell fate through YAP/TAZ (Fig. 8).

How YAP/TAZ and mechanical signals mediate their potent biological effects by controlling transcription is a critical, albeit still enigmatic, issue. Here we uncovered a surprising Notch-inhibitory effect of YAP/TAZ activation. In so doing, YAP/TAZ serve as nexus to translate mechanical signals into mechanical control of Notch signalling (Fig. 8). YAP/TAZ directly regulate the expression of Delta-like ligands that are essential to orchestrate Notch signalling in epidermal progenitors. Indeed, Notch receptors are activated by Delta ligands when these are expressed by different neighbouring cells, but these receptors are inhibited when the same ligands are co-expressed in the same cell. We propose that YAP/TAZ, by regulating the transcription of ligands such as DLL1 in epidermal SCs, maintain them in an undifferentiated state through *cis*-inhibition. In so doing, YAP/TAZ coordinate informational cues emanating from cell–ECM attachment into a cell–cell signalling pathway controlling fine-grained, mutual regulation of opposing cell fate decisions. The paradigm here identified in epidermal SCs may apply to other contexts. For example, YAP/TAZ are basally expressed in other stratified epithelia, such as during development of the mammary gland^48^ and airways^49^; these compartments also contains SCs and progenitors that also must escape the differentiating effects of Notch signalling to preserve their identity^50^,^51^.

YAP/TAZ are instrumental and essential for skin tumorigenesis, for which Notch is a tumour suppressor^19^,^33^,^52^. Coupling YAP/TAZ mechanotransduction to Notch in epidermis may thus represent a fail-safe mechanism to limit skin tumorigenesis by confining tumour-initiating cells only to appropriate, permissive mechanical niches in the basal layer, while inducing differentiation of cells exiting such niches by unleashed Notch signalling. Consistent with this idea and with the established role of YAP/TAZ and Notch signalling in skin carcinogenesis^26^,^33^,^36^, we also found that YAP/TAZ and Notch transcriptional signatures are anticorrelated in skin tumour samples (mouse skin chemical carcinogenesis or human skin squamous cell carcinoma cell lines versus their normal counterpart; Supplementary Fig. 4b,c).

The biological effects of Notch signalling are highly context dependent, triggering proliferation or terminal differentiation in different cell types. In several tumour types, YAP/TAZ and Notch signalling are both considered potent oncogenes^36^,^53^, but few studies have addressed the effects of these signals in distinct tumour cell subpopulations. In fact, the YAP/TAZ-Notch connection here described may be also compatible with a scenario in which YAP/TAZ, known for being expressed in cancer stem cells^36^, may preserve their undifferentiated state by *cis*-inhibition of Notch, and, at the same time, induce proliferation of neighbouring tumour cells by *trans*-activation of Notch signalling.

Finally, a number of inborn and acquired disorders, as well as aging, actually affect tissue mechanics by modifying ECM, adhesive and cytoskeletal proteins, leading to loss or exhaustion of tissue SCs^54^,^55^. Our findings bear implications for the use of YAP/TAZ^56^, of appropriate substrate mechanics/topology or direct manipulation of Notch signalling to expand normal somatic epidermal SCs *ex vivo*, possibly opening innovative routes for understanding and treating these conditions.

## Methods

### Cell line and transfection

Primary human epidermal keratinocytes isolated from neonatal foreskin (nHEK) were provided and characterized by ThermoFisher (C0015C). Cells were cultured and expanded in low calcium, antibiotics-free and serum-free EpiLife Medium (ThermoFisher MEPI500CA), supplemented with human keratinocyte growth supplement (ThermoFisher S0015), according to the manufacturer's instructions. The nHEK cells and the culture conditions were mycoplasma-free. Low-passage (p3–p4), exponentially growing nHEK cells were used for all the experiments. siRNAs were transfected with Lipofectamine RNAi-MAX (ThermoFisher); DNA was transfected with Lipofectamine LTX PLUS Reagent (ThermoFisher). All transfections were performed in EpiLife Medium, according to the manufacturer's instructions. Unless otherwise indicated, siYAP/TAZ is mix siYAP/TAZ #1. Sequences of all siRNAs are provided in Supplementary Data 2.

### Microfabrications and experimental settings

Micropatterned glass slides were purchased from Cytoo SA. Cytoo chips consist of 20 × 20 mm^2^ coverslips with an organized grid of fibronectin-coated micropatterns on the top. PADO2-SQ17 code identifies chips containing only square islands of 300 μm^2^ (ref. 21); Custom Square 100222 code stands for chips containing islands of 300; 1,000; 2,000 and 10,000 μm^2^ arrayed in quadrants^22^. Fibronectin-coated hydrogels of different elastic moduli (0.7–80 kPa) were synthesized as previously described^22^. For experiments with hydrogels, cells were seeded in a 400 μl drop at the centre of the dish; after attachment, the wells containing the hydrogels were filled with medium. For stiff versus soft experiments, cells were plated, respectively, at 2,000 cells cm^−2^ for IF and at 10,000 cells cm^−2^ for qPCR assays. For assays on micropatterned glass slides, 150,000 cells were plated in a 35 mm dish containing a single Cytoo glass slide and nonadherent cells were washed with medium after 2 h. For experiments with different cell densities, cells were plated at 5,000 cells cm^−2^ to obtain low-density cultures (sparse) or at 200,000 cells cm^−2^ to obtain monolayers at postconfluent cell density (dense).

### Mice

Transgenic lines used in the experiments were kindly provided by: Silvio Gutkind (*K5-rtTA*)^57^; Fernando Camargo (*tetO-YAP*^*S127A*^)^58^; Pierre Chambon (*K14-CreER*^*T2*^)^59^; and Doujia Pan (*Yap*^*fl/fl*^)^60^. *Taz*^*fl/fl*^ mice were as in ref. 61. Animals were genotyped with standard procedures and with the recommended set of primers. Animal experiments were performed adhering to our institutional guidelines as approved by OPBA (University of Padova) and the Italian Ministry of Health.

To obtain *K5-rtTA; tetO-YAP*^*S127A*^ mice, we crossed *K5-rtTA* hemizygous females with *tetO-YAP*^*S127A*^ heterozygote males. Starting at E14.5 or E16.5 from vaginal plugs, pregnant females were administered doxycycline (2 mg ml^−1^ in their drinking water supplemented with 10 mg ml^−1^ sucrose) to induce transgene expression. After 96 h, pregnant females were sacrificed and embryos at E18.5 or E20.5, respectively, were harvested. Genotypes were confirmed on embryos biopsies and skin samples were processed for further analyses. *K5-rtTA* littermates were used as normal controls.

To obtain *K14-CreER*^*T2*^*; Yap*^*fl/fl*^; *Taz*^*fl/fl*^ mice, we crossed *K14-CreER*^*T2*^*; Yap*^*fl/fl*^; *Taz*^*fl/fl*^ males with *Yap*^*fl/fl*^; *Taz*^*fl/fl*^ females. Starting at E13.5 from vaginal plugs, pregnant females were injected with Tamoxifen (Sigma) to induce recombination in the embryos. Pregnant females were sacrificed after 96 h from Tamoxifen injection and embryos at E17.5 were harvested. Genotypes were confirmed on embryos biopsies and skin samples of K14-CreER-positive and their control littermates were processed for further analyses.

### YAP/TAZ peaks' annotation

We created a database of YAP/TAZ-binding regions in the human genome using ChIP-seq data for YAP and TAZ from refs 33, 62. YAP or TAZ peaks from individual ChIP-seq analyses from three different cell lines (MDA-MB-231, SF268, NCI-H2052) were combined in a single list of YAP/TAZ peaks, representing a general reference list for YAP/TAZ-binding sites in human cells. YAP/TAZ peaks were annotated as falling on promoters if they were close to a TSS (±2 kb); otherwise they were annotated as located in enhancers. YAP/TAZ peaks falling on promoters were assigned to the closest TSS. YAP/TAZ peaks falling on enhancers were annotated using the chromatin interactions looping to promoters reported in Supplementary Data 1 of ref. 63 and the promoter–enhancer interaction data set (Array Express E-MTAB-2323) from ref. 64, derived from high-resolution Hi-C and high-resolution capture Hi-C experiments, respectively. Each data sheet reports the genomic locations of all target peaks interacting with a list of >10,000 anchors located at gene promoters. YAP/TAZ peaks overlapping with these target peaks were assigned to the corresponding interacting promoter region. See Supplementary Data 1 for the list of YAP/TAZ annotated peaks.

### Reagents and plasmids

Doxycycline hyclate, latrunculin A, D-luciferin, DAPT and DBZ were from Sigma; fibronectin was from Santa Cruz Biotechnology; cytochalasin D was from Calbiochem; CPRG was from Roche. For the inducible expression of YAP constructs, cDNA for human YAP1 5SA (LATS-mutant sites)^21^ and human YAP1 5SA/S94A (TEAD-binding mutant)^34^ were made insensitive to YAP siRNA #1 and subcloned, together with an HA-tag, in FUW-tetO-MCS, obtained by substituting the Oct4 sequence in FUW-tetO-hOct4 (Addgene #20726)^65^ with a new multiple cloning site (MCS). This generated FUW-tetO-HA-YAP5SA and FUW-tetO-HA-YAP5SA/S94A were used throughout this study. FUW-tetO-MCS (empty vector) or FUW-tetO-RFP plasmids, obtained by subcloning the RFP coding sequence from the pTomo vector (Addgene #26291), were used as controls. FUdeltaGW-rtTA was from Addgene (#19780)^66^. FUW-tetO-N1ICD-Myc and FUW-tetO-GFP-DNMAML1 were obtained by subloning in the FUW-tetO-MCS the corresponding coding sequences, respectively, from pcDNA3 N1ICD-Myc^67^ and MigRI-DNMAML1-GFP^38^. All constructs were confirmed by sequencing. The 8xGTIIC-lux (Addgene #34615) was previously described^22^. The 3D.A-Lux was gently provided by Guido Posern^68^. Primers for RT–PCR are listed in Supplementary Data 3.

### Lentivirus preparation

HEK293T cells (checked routinely for the absence of mycoplasma contaminations) were kept in DMEM supplemented with 10% FBS, 1% Glutamine and 1% Pen/Strep antibiotics (ThermoFisher). Lentiviral particles were prepared by transiently transfecting HEK293T with lentiviral vectors together with packaging vectors pMD2-VSVG and pPAX2 by using TransIT-LT1 (Mirus Bio) according to the manufacturer's instructions. For the collection of viruses for keratinocytes infection, DMEM medium was washed out 8 h after transfection and Epilife Medium supplemented with human keratinocyte growth supplement was added. Supernatant was collected 48 h after transfection.

### Chromatin immunoprecipitation-qPCR

ChIP was performed as previously described^33^. Briefly, cells were crosslinked with 1% formaldehyde (Sigma) in culture medium for 10 min at room temperature, and chromatin from lysed nuclei was sheared to 200–600 bp fragments using a Branson Sonifier 450 A. For ChIP-qPCR, ∼100 μg of sheared chromatin and 3–5 μg of antibody were used. Antibody/antigen complexes were recovered with ProteinA-Dynabeads (ThermoFisher) for 2 h at 4 °C. Antibodies are listed in Supplementary Data 4. Where indicated, cells were treated with latrunculin-A 0.8 μM for 4 h before processing for ChIP. Quantitative real-time PCR was carried out with QuantStudio 5 thermal cycler (ThermoFisher); each sample was analysed in triplicate. The amount of immunoprecipitated DNA in each sample was determined as the fraction of the input (amplification efficiency^(Ct INPUT−Ct ChIP)^) and normalized to the immunoglobulin G control. Primers are listed in Supplementary Data 5.

### Reproducibility of experiments and statistical analysis

For qRT–PCR on primary cells, at least three independent experiments (each with at least two biological replicates and three technical replicas for each biological replicate) were performed with similar results. Indeed, a second independent experiment is shown in Supplementary Fig. 6 for each of the panels shown in the main figures. If not otherwise indicated, data from two biological replicates (mean+s.d.) from one representative experiment are shown. For experiments on skin biopsies, at least three independent experiments (each with at least two biological replicates and three technical replicas for each biological replicate) were performed; animal ages are specified in the text and Methods section. For IF and immunohistochemistry, at least three independent experiments were analysed; for IF analysis on primary cells, at least 100 cells for each condition were scored as described. Western blots were performed at least three times with similar results. For luciferase assays, each experiment contained two biological replicates and was repeated at least three times independently. Statistical analyses were performed with GraphPad Prism 7. Differences at *P*≤0.05 were considered statistically significant. *P* values were calculated by analysis of variance for multiple pairwise comparisons or paired two-tail Student's *t*-test for comparisons of two groups. All the experiments were performed without methods of randomization or blinding and the sample size was not predetermined.

### Data availability

The data that support the findings of this study are available from the corresponding author upon request.

## Additional information

**How to cite this article:** Totaro, A *et al*. YAP/TAZ link cell mechanics to Notch signalling to control epidermal stem cell fate. *Nat. Commun.* **8**, 15206 doi: 10.1038/ncomms15206 (2017).

**Publisher's note:** Springer Nature remains neutral with regard to jurisdictional claims in published maps and institutional affiliations.

## Supplementary Material

Supplementary Information Supplementary Figures, Supplementary Methods and Supplementary References

Supplementary Data 1 Full list of YAP/TAZ binding sites in the human genome derived from ChIP-seq analyses from MDA-MB-231, SF268 and NCI-H2052 cell lines. (A) Peak ID. (B-D) Genomic coordinates of each peak. (E-F) Each YAP/TAZ peak is also identified with the corresponding peak ID from the TAZ ChIP-seq in MDA-MB-231 (Zanconato et al., 2015) and from the YAP ChIP-seq in SF268 and NCI-H2052 cell lines (Stein et al., 2015). NA indicates that no YAP/TAZ peaks were identified at a specific genomic region in a specific cell line. (H) Name of the gene associated with the TSS closest to each peak. (I) Distance of each peak to the closest TSS. (J) Classification of each peak as located in a promoter (1), or in an enhancer (0). (K-L) Peaks on enhancers were assigned to target genes based on enhancer-promoter interactions from a high resolution Hi-C study on IMR90 (K), and from high resolution capture Hi-C study on GM12878 (L). NA indicates that a given peak could not be matched with any enhancer-promoter pairs.

Supplementary Data 2 Sequence of the siRNAs.

Supplementary Data 3 Sequence of primers for qRT-PCR.

Supplementary Data 4 List of antibodies and their working conditions.

Supplementary Data 5 Sequence of primers for Chip-PCR.
