# Supplementary material for: YAP/TAZ link cell mechanics to Notch signalling to control epidermal stem cell fate
Source: Nat Commun. 2017 May 17;8:15206. doi: 10.1038/ncomms15206 (PMC5442321; doi:10.1038/ncomms15206)
Supplement: Supplementary Information — Supplementary Figures, Supplementary Methods and Supplementary References [file ncomms15206-s1.pdf]

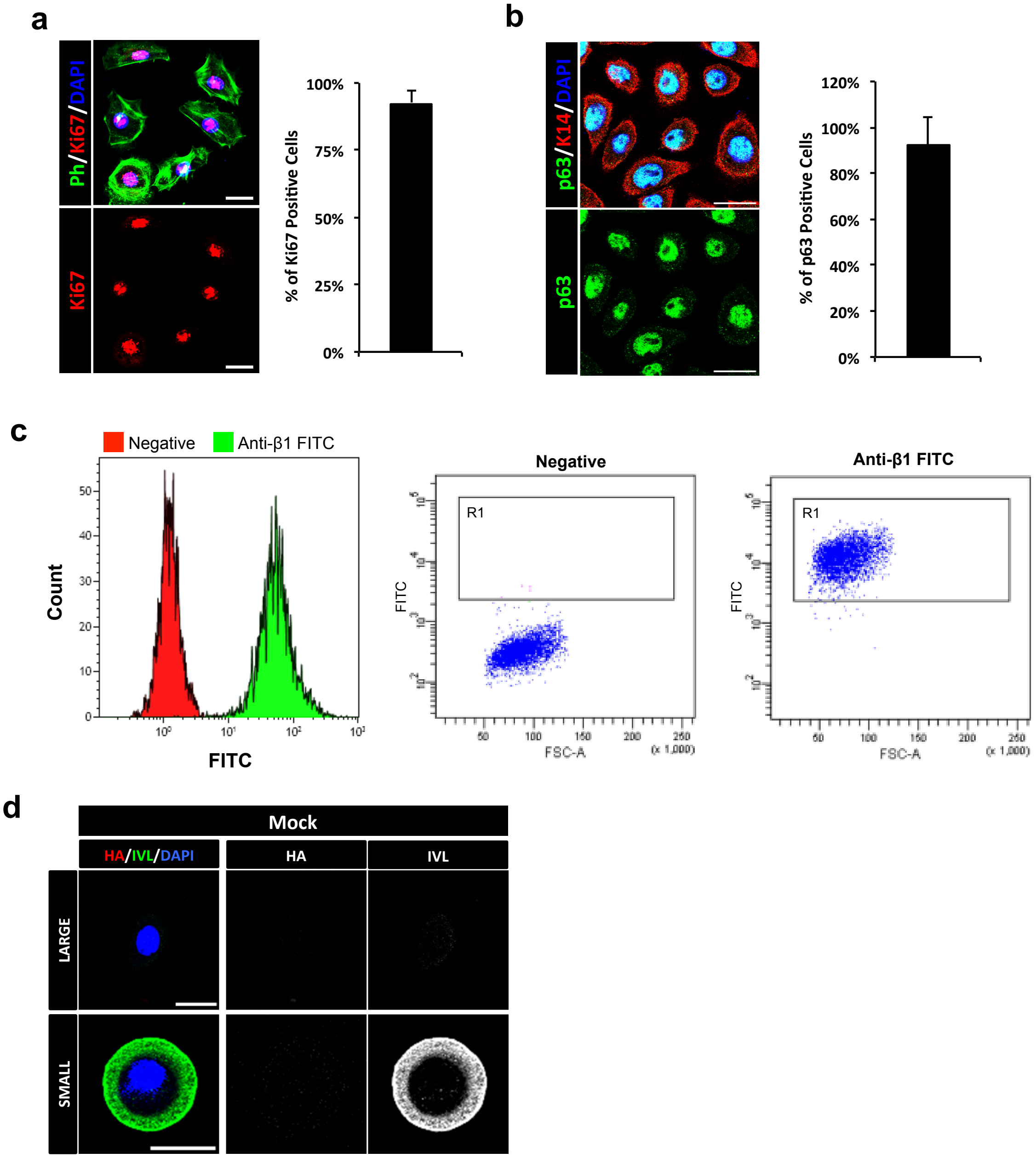
a
Ph/Ki67/DAPI
Ki67
% of Ki67 Positive Cells
100%
75%
50%
25%
0%
b
p63/K14/DAPI
p63
% of p63 Positive Cells
120%
100%
80%
60%
40%
20%
0%
c
Negative
Anti-β1 FITC
Count
FITC
Negative
Anti-β1 FITC
R1
FITC
FSC-A
(x 1,000)
d
Mock
HA/IVL/DAPI
HA
IVL
LARGE
SMALL

Supplementary Figure 1 Characterization of Neonatal Human Epidermal Keratinocytes (nHEK).

(**a**) Confocal immunofluorescence images of low-passage nHEK cells (p3) stained for the Ki67 proliferation marker (red) and the cytoskeletal F-actin (Phalloidin, green). DAPI (blue) is the nuclear counterstain. Scale bar: 20 μm. Human keratinocytes, expanded in vitro according to the manufacturer's instruction, are highly proliferating as shown by the percentage (95±5%) of Ki67-positive cells. Bars represent mean + SD (n = 3 independent experiments).

(**b**) Confocal immunofluorescence images of nHEK cells stained for the p63 keratinocyte stem marker (green) and the basal keratin 14 (K14, red). Scale bar: 20 μm. nHEK cultures are highly enriched of epidermal stem cells since most of the cells (92±12%) express high levels of nuclear p63. Bars represent mean + SD (n = 3 independent experiments).

(**c**) nHEK cells (p3) were stained with a FITC-conjugated anti-β1 integrin antibody and analysed by Flow cytometry. The histogram plots the FITC intensity (horizontal axis) against the number of events detected (vertical axis). Cells stained with anti-β1 integrin are plotted in green, negative control cells are plotted in red. Flow cytometry plots for forward scatter (FS) versus FITC channel for negative- (non-labelled) and β1 integrin-labelled cells. R1: gating for β1 integrin expression. More than 95% of nHEK cells were positive for β1 integrin staining.

(**d**) nHEK cells were infected with an empty vector (Mock) and treated as in Fig 1f. Confocal images show representative staining for HA-tag in Mock-infected cells (red), merged with the Involucrin differentiation marker (green) and the nuclear DAPI (blue) (right panel). The same images are shown as single channel in grayscale (left panel) as control of the background staining. Scale bar: 20 μm.

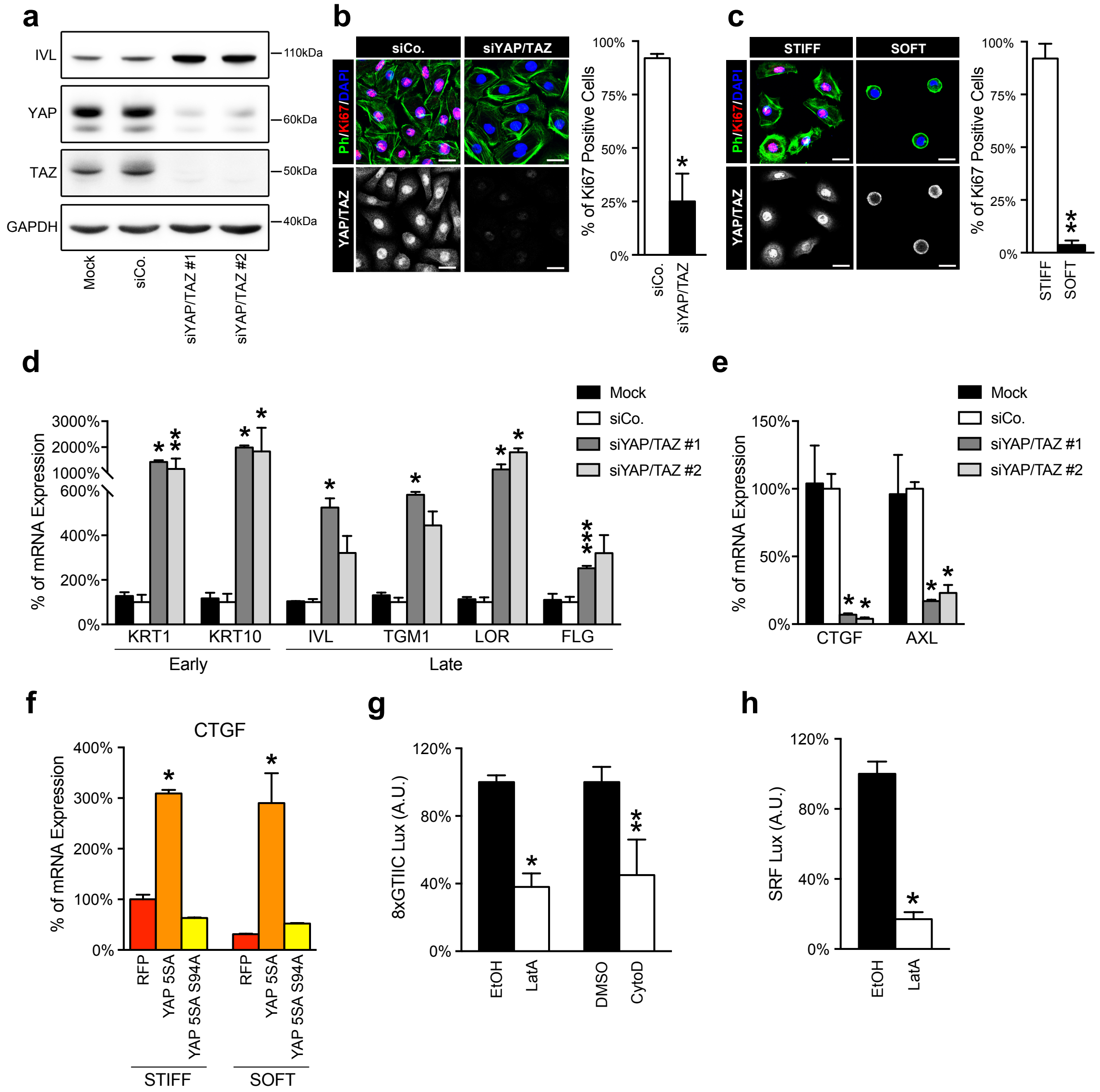
a
IVL
YAP
TAZ
GAPDH
110kDa
60kDa
50kDa
40kDa
Mock
siCo.
siYAP/TAZ #1
siYAP/TAZ #2
b
siCo.
siYAP/TAZ
Ph/Ki67/DAPI
YAP/TAZ
% of Ki67 Positive Cells
c
STIFF
SOFT
d
% of mRNA Expression
KRT1
KRT10
IVL
TGM1
LOR
FLG
Early
Late
Mock
siCo.
siYAP/TAZ #1
siYAP/TAZ #2
e
CTGF
AXL
f
CTGF
RFP
YAP 5SA
YAP 5SA S94A
g
8xGTIIC Lux (A.U.)
EtOH
LatA
DMSO
CytoD
h
SRF Lux (A.U.)

Supplementary Figure 2 Mechanical signals control the fate of epidermal SCs through YAP/TAZ.

(**a**) Representative Western Blots for Involucrin (IVL), YAP, TAZ and GAPDH proteins from lysates of nHEK cells transfected with a mock solution (Mock), control siRNA (siCo.) or two independent sets of siRNAs targeting both YAP and TAZ mRNAs. YAP/TAZ knockdown increases the expression of the Involucrin protein. The YAP and TAZ staining confirms the effectiveness of silencing for the siYAP/TAZ mixes. GAPDH serves as loading control. See Methods for reproducibility of experiments.

(**b-c**) nHEK cells were transfected as in Fig. 1d (**b**) or plated either on fibronectin-coated coverslips (STIFF) and 1 kPa fibronectin-coated polyacrylamide hydrogels (SOFT) (**c**). Panel shows confocal images of a quadruple staining for Ki67 (red), cytoskeletal F-actin (Phalloidin, green) and nuclear DAPI (blue) (merged, upper panel); the staining for the endogenous YAP/TAZ proteins is shown separately (grey scale, lower panel). Terminal differentiation of keratinocytes induced by inhibition of YAP/TAZ activity, either by siRNA knockdown (**b**) or by Soft ECM (**c**), is associated with cell cycle exit as confirmed by the reduction of the expression of the cell cycle related protein Ki67 and quantified in the chart as percentage of nuclear Ki67 positive cells. Bars represent mean + SD (n = 3 independent experiments. * P < 0,001 compared to siCo., ** P < 0,0001 compared to Stiff; Student's *t*-test).

(**d-e**) qRT-PCR analysis of nHEK cells transfected as in **a**. Values were normalized to siCo.-treated cells for each gene analysed (white bars). (**d**) qRT-PCR of Early [*KRT1, KRT10*] and Late [*IVL*, *TGM1*, *LOR, FLG*] keratinocyte differentiation genes confirms that YAP/TAZ knockdown instructs a general programme of terminal differentiation of epidermal keratinocytes. (**e**) qRT-PCR analysis of mRNA levels of *CTGF* and *AXL* target genes confirms downregulation of the YAP/TAZ transcriptional activity upon siRNA transfection. Bars represent mean + SD (* P < 0.001, ** P < 0.01, *** P < 0.05 compared to siCo.; Student's *t*-test).

(**f**) nHEK cells were infected with the indicated doxycycline-inducible lentiviral constructs and replated for 24 hours on Stiff or Soft conditions. YAP/TAZ transcriptional activity was evaluated by qRT-PCR on the *CTGF* target gene. Values were normalized to RFP-infected cells plated on Stiff. When compared to cells seeded on Stiff, cells seeded on a Soft substrate downregulated the expression of the *CTGF* YAP/TAZ target gene. However the expression of *CTGF* in cells plated on a Soft ECM can be restored by the transduction of an active form of YAP (YAP5SA), but not by the transcriptionally deficient YAP mutant (YAP5SA/S94A). Bars represent mean + SD (* P < 0.01, compared to the corresponding RFP-control condition; one way ANOVA). (**d-f**) See Methods for reproducibility of experiments.

(**g**) Luciferase assay for YAP/TAZ dependent transcriptional activity in nHEK cells upon treatment with F-actin inhibitors. Cells were transfected with 8xGTIIC-Lux reporter and treated with either vehicle (EtOH) and 0.8 μM latrunculin A (LatA) or with vehicle

(DMSO) and 2.5 μM cytochalasin D (CytoD). Data were normalized to the corresponding controls and presented in arbitrary units (A.U.) as mean + SD (n = 3 independent experiments. * P = 0.0014 LatA, ** P = 0.03 CytoD; Student's *t*-test).

(**h**) Luciferase assay for SRF dependent transcriptional activity in nHEK cells upon treatment with F-actin inhibitors. Cells were transfected with SRF 3D.A-Lux reporter and treated with vehicle (EtOH) or 0.8 μM latrunculin A (LatA). Data were normalized to the vehicle control and presented in arbitrary units (A.U.) as mean + SD (n = 3 independent experiments. * P < 0,001; Student's *t*-test).

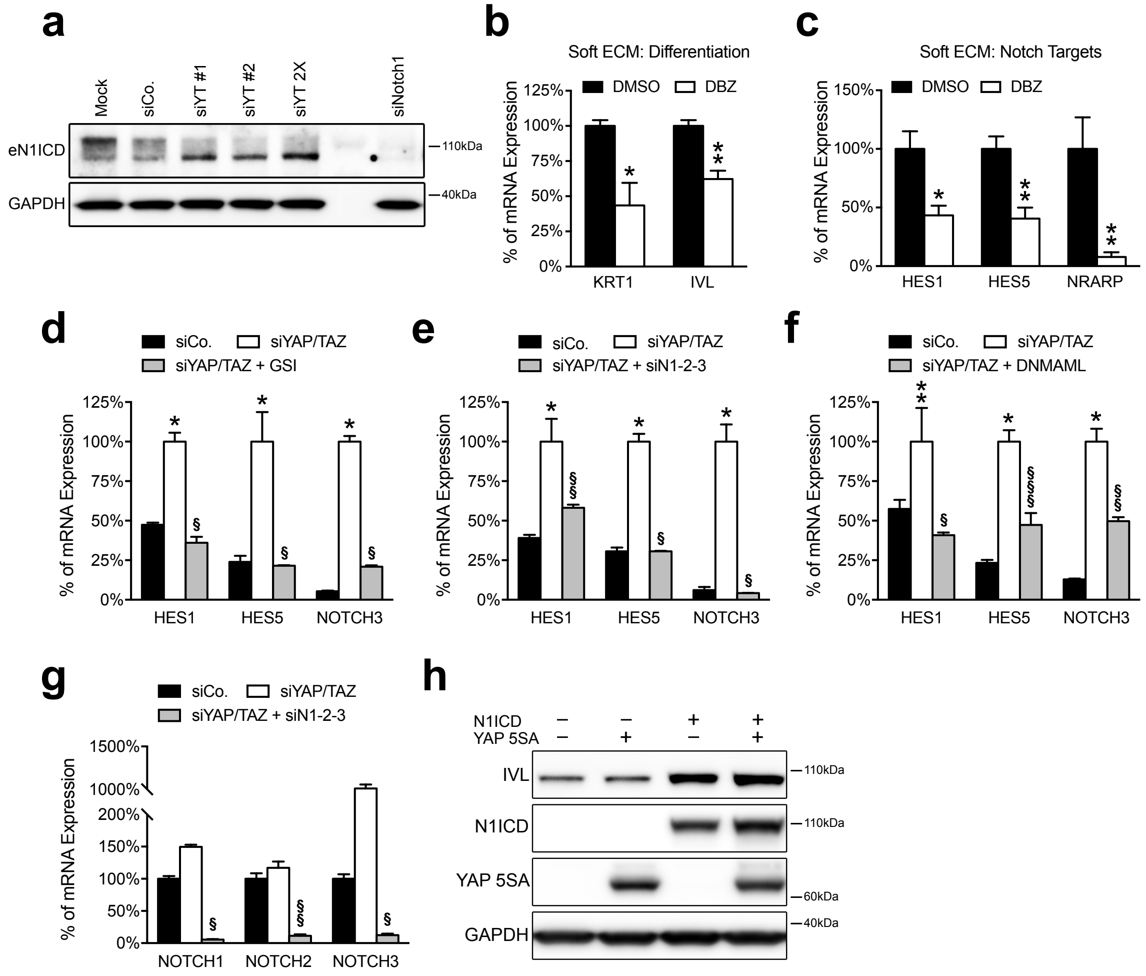

a
Mock
siCo.
siYT #1
siYT #2
siYT 2X
siNotch1
eN1ICD
GAPDH
110kDa
40kDa
b
Soft ECM: Differentiation
DMSO
DBZ
% of mRNA Expression
KRT1
IVL
c
Soft ECM: Notch Targets
HES1
HES5
NRARP
d
siCo.
siYAP/TAZ
siYAP/TAZ + GSI
HES1
HES5
NOTCH3
e
siYAP/TAZ + siN1-2-3
f
siYAP/TAZ + DNMAML
g
NOTCH1
NOTCH2
NOTCH3
h
N1ICD
YAP 5SA
IVL
N1ICD
YAP 5SA
GAPDH
110kDa
60kDa
40kDa

Supplementary Figure 3 YAP/TAZ regulate epidermal SC differentiation through Notch signaling.

(**a**) nHEK cells plated in mechanically stiff conditions were transfected with the indicated siRNAs and after 48 hours analysed by western blot for the presence of the active form of the Notch1 intracellular domain (N1ICD). Knock down of YAP/TAZ with three different siRNA mixes increases the levels of the transcriptionally active fragment of the Notch1 receptor (N1ICD) (siYT 2x is combination of siYT #1 e siYT #2, siNotch1 is used as control of band specificity). See Methods for reproducibility of experiments.

(**b-c**) Notch activity is required for mechano-induced keratinocyte differentiation. nHEK cells plated on fibronectin-coated hydrogels of 1 kPa (SOFT) and treated with control DMSO or DBZ (2.5 μM) were analysed by qRT-PCR for the expression of the *KRT1* and *IVL* differentiation markers (**b**) and for the Notch target genes *HES1*, *HES5*, *NRARP* (**c**). Data were normalized to the DMSO treated cells for each gene analysed. Bars represent mean + SD (n = 4. * P < 0.001, ** P =< 0.0001 compared to respective DMSO treated controls; Student's *t*-test).

(**d-f**) Validation of Notch signaling inhibition upon treatment of YAP/TAZ-silenced keratinocytes with either GSI (**d**), transfection of anti-Notch 1-3 siRNAs (siN1-2-3) (**e**) or DNMAML (**f**) as previously described (Fig. 5c-e). nHEK cells were analysed by qRT-PCR for the expression the Notch target genes *HES1*, *HES5*, *NOTCH3*. Data were normalized to the control-treated siYAP/TAZ transfected cells (white bar) for each gene analysed.

(**g**) Validation of Notch receptors knockdown after siRNAs transfection against Notch 1-2-3, as in Fig. 5d and Supplementary Fig. 3d. nHEK cells were analysed by qRT-PCR for the expression the Notch receptor *NOTCH1*, *NOTCH2* and *NOTCH3*. Data were normalized to the control-transfected cells (siCo.) for each gene analysed. (**d-g**) Bars represent mean + SD (* P < 0.0001, ** P =< 0.001, compared siCo.; § P < 0.0001, §§ P =< 0.001; §§§ < 0.05, compared to siYAP/TAZ; two-way ANOVA). See Methods for reproducibility of experiments.

(**h**) nHEK cells infected with an empty construct or with the indicated combination of lentiviral vectors encoding for an activated form of YAP (YAP5SA) or for the active Notch1 intracellular domain (N1ICD) were analysed by western blot for the expression of the Involucrin (IVL) differentiation marker. GAPDH serves as loading control. The YAP and N1ICD staining, on corresponding amount of lysates loaded on a different gel, confirm the efficacy of the infection. See Methods for reproducibility of experiments.

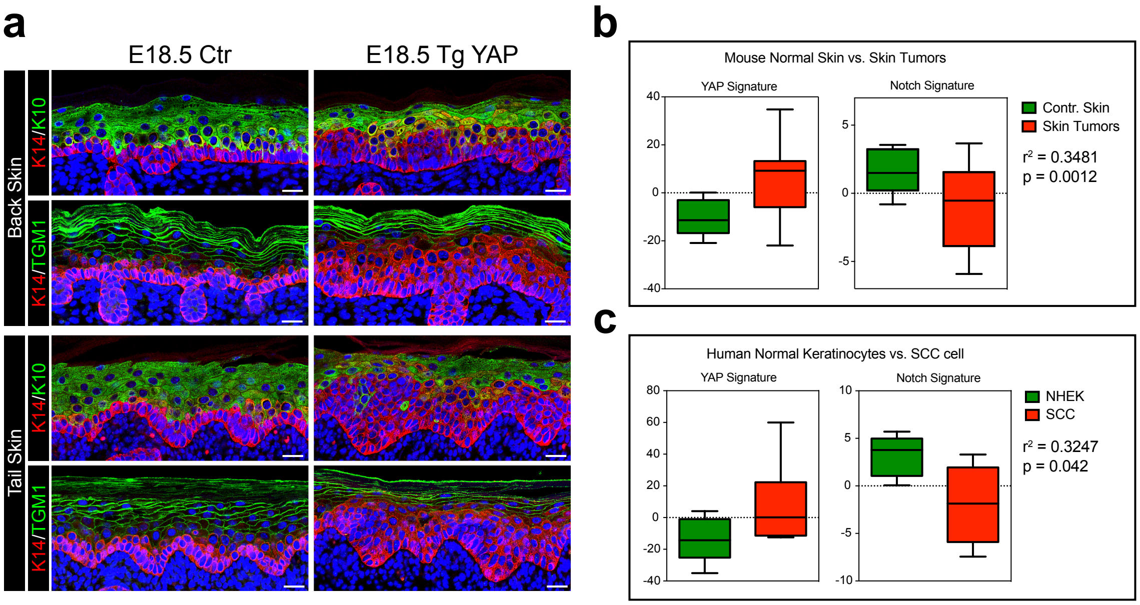

Supplementary Figure 4 In vivo validation of YAP/TAZ as inhibitors of Notch signaling in epidermis.

(**a**) Immunofluorescence on the back- and tail-skin of E18.5 embryos as is Fig. 6a. YAP induction increases the thickness of the KRT14-positive basal layers at the expense of the differentiated KRT10- and TGM1-positive suprabasal layers. Tg YAP: YAP-transgenic mice, Ctr: control littermates; K14: keratin 14; K10: keratin 10. Scale bar: 20 μm.

(**b-c**) Box plots of the distributions of the Z-scores for gene expression signatures of YAP and NOTCH activities. Samples are from published gene expression profiles of normal mouse dorsal skin samples (Contr. Skin) and skin carcinomas arising after application of the chemical carcinogenesis protocol on mouse skin (Skin Tumors) (**b**), or normal human keratinocytes (NHEK) and Squamous Cell Carcinoma human cell lines (SCC) (**c**). In the different panels, statistical significances between distributions were assayed using two-tailed t-test with Welch's correction for different variances.

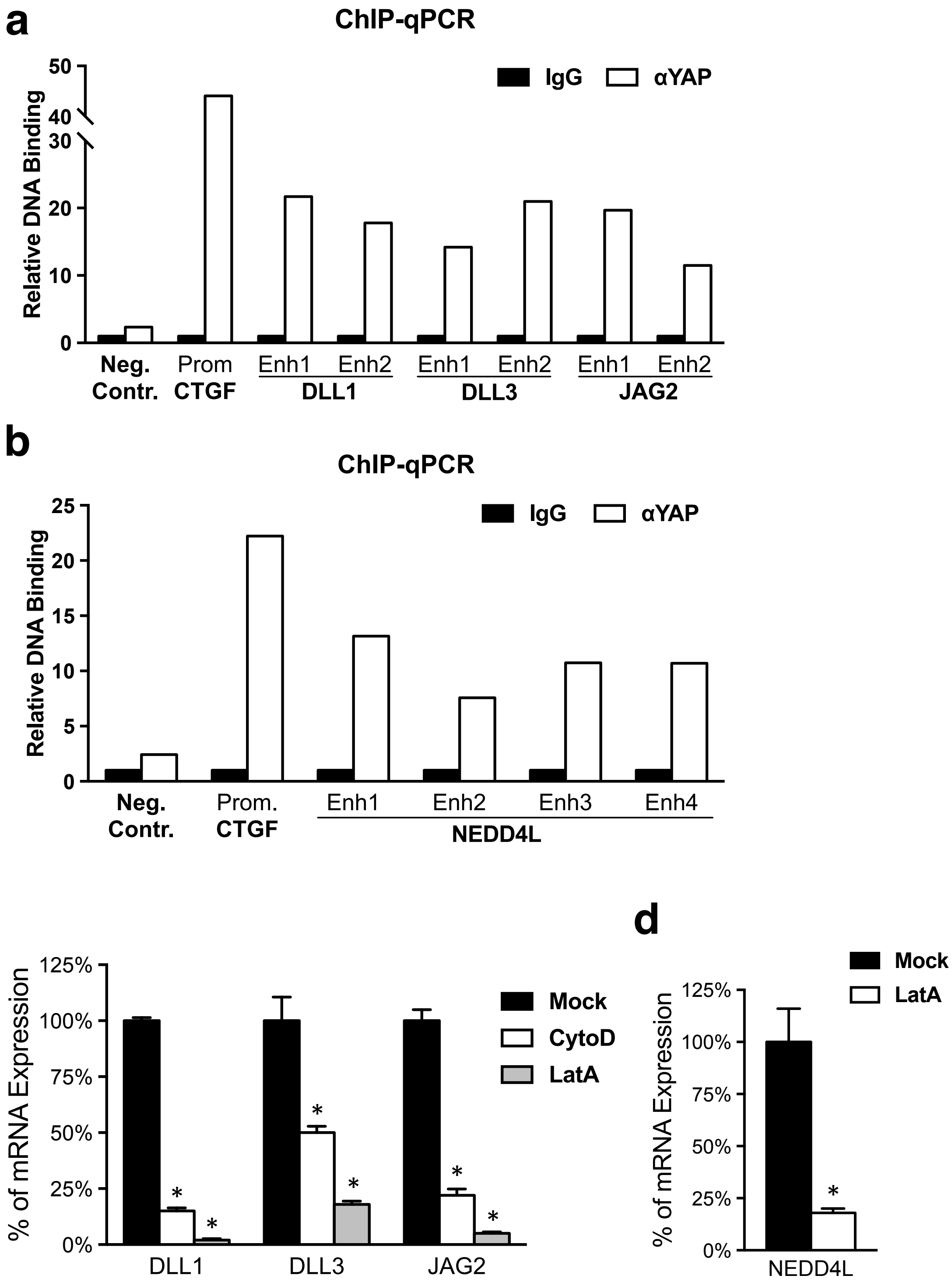
a
ChIP-qPCR
Relative DNA Binding
0
10
20
30
40
50
IgG
αYAP
Neg. Contr.
Prom CTGF
Enh1
Enh2
DLL1
Enh1
Enh2
DLL3
Enh1
Enh2
JAG2
b
ChIP-qPCR
Relative DNA Binding
0
5
10
15
20
25
IgG
αYAP
Neg. Contr.
Prom. CTGF
Enh1
Enh2
Enh3
Enh4
NEDD4L
c
% of mRNA Expression
0%
25%
50%
75%
100%
125%
Mock
CytoD
LatA
*
DLL1
DLL3
JAG2
d
Mock
LatA
% of mRNA Expression
0%
25%
50%
75%
100%
125%
*
NEDD4L

Supplementary Figure 5 YAP/TAZ transcriptionally control Notch regulators.

(**a-b**) ChIP-qPCR verifying YAP binding to the enhancers associated with the Notch signaling regulators DLL1, DLL3, JAG2 (**a**) and NEDD4L (**b**). *DLL1*, *DLL3*, *JAG2* and *NEDD4L* enhancer sequences were enriched in YAP-immunoprecipitated chromatin from nHEK cells, but not in negative control IP (IgG). *CTGF* promoter is a positive control locus; *HBB* is a negative control locus (Neg Contr.). Relative DNA binding was calculated as fraction of input and normalized to IgG. Data from one representative experiment out of three are shown.

(**c**) nHEK cells treated for 24 hours with control vehicle (Mock), 2.5 μM cytochalasin D (CytoD) or 0.8 μM latrunculin A (LatA) were analysed by qRT-PCR for the expression of the Notch ligands *DLL1*, *DLL3* and *JAG2*. Values were normalized to the Mock-treated cells. Bars represent mean + SD (n = 3 independent experiments. * $P \leq 0{,}0001$ compared to Mock; two-way ANOVA)

(**d**) nHEK cells treated for 24 hours with control vehicle (Mock) or 0.8 μM latrunculin A (LatA) were analysed by qRT-PCR for the expression of *NEDD4L*. Values were normalized to the Mock-treated cells. Bars represent mean + SD (n = 3 independent experiments, * $P = 0{,}0015$ compared to Mock; one-way ANOVA).

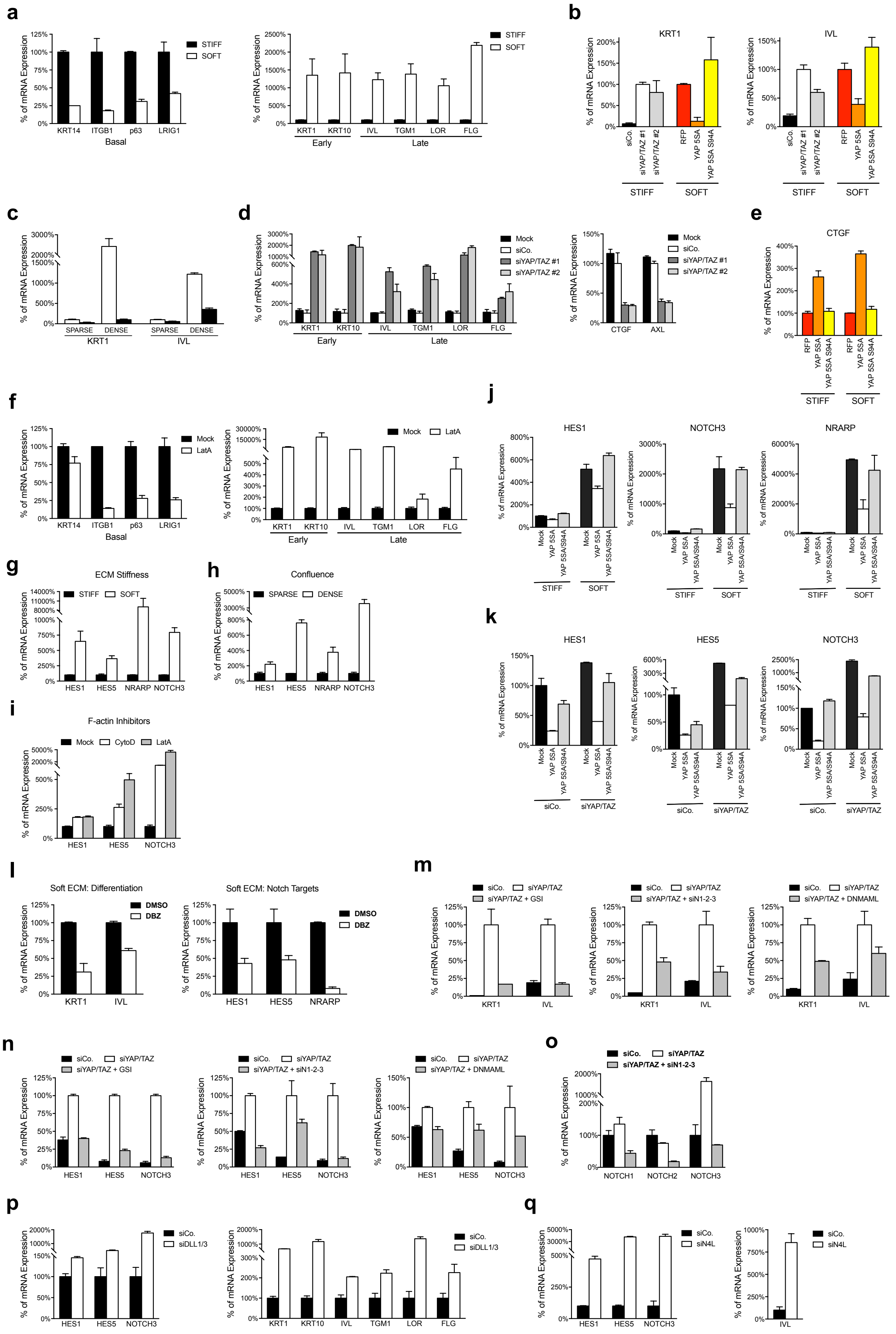
a
% of mRNA Expression
STIFF
SOFT
KRT14
ITGB1
p63
LRIG1
Basal
KRT1
KRT10
Early
IVL
TGM1
LOR
FLG
Late
b
KRT1
IVL
siCo.
siYAP/TAZ #1
siYAP/TAZ #2
RFP
YAP 5SA
YAP 5SA S94A
STIFF
SOFT
c
SPARSE
DENSE
KRT1
IVL
d
Mock
siCo.
siYAP/TAZ #1
siYAP/TAZ #2
Early
Late
CTGF
AXL
e
CTGF
f
Mock
LatA
g
ECM Stiffness
HES1
HES5
NRARP
NOTCH3
h
Confluence
i
F-actin Inhibitors
CytoD
j
HES1
NOTCH3
NRARP
YAP 5SA/S94A
k
HES5
siYAP/TAZ
l
Soft ECM: Differentiation
Soft ECM: Notch Targets
DMSO
DBZ
m
siYAP/TAZ + GSI
siYAP/TAZ + siN1-2-3
siYAP/TAZ + DNMAML
n
o
NOTCH1
NOTCH2
p
siDLL1/3
q
siN4L

Supplementary Figure 6 Replica of qRT-PCR data.
A second independent experiment is shown for each of the panels presented in the Main Figures. (**a**) Replica of Fig. 2d,e; (**b**) replica of Fig. 2f; (**c**) replica of Fig. 2j; (**d**) replica of Supplementary Fig. 2d,e; (**e**) replica of Supplementary Fig. 2f; (**f**) replica of Supplementary Fig. 3d,e; (**g-i**) replica of Fig. 4a-c; (**j,k**) replica of Fig. 4d,e; (**l**) replica of Supplementary Fig. 3b,c; (**m**) replica of Fig. 5c-e; (**n,o**) replica of Supplementary Fig. 3d-g; (**p**) replica of Fig. 7d,e; (**g**) replica of Fig. 7h,i.

Supplementary Figure 2a

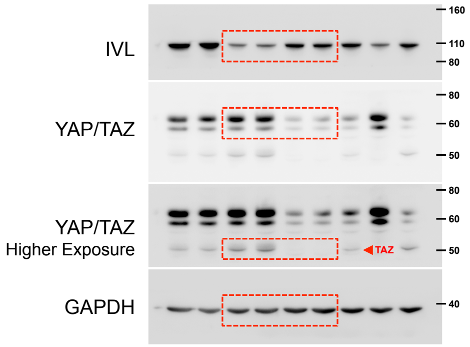

Supplementary Figure 3h

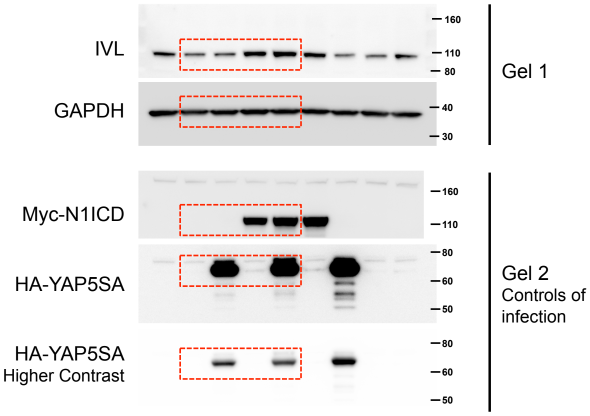

Supplementary Figure 3g

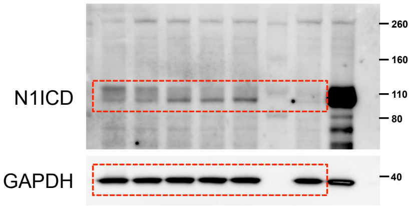

Supplementary Figure 7 Uncropped Western blots.
Uncropped images of immunoblots displayed in the figures. Dashed boxes indicate areas that were cropped.

SUPPLEMENTARY METHODS

Luciferase Assays. Luciferase YAP/TAZ reporter 8xGTIIC-Lux or SRF reporter 3D.A Lux (150 ng/cm^2) were transfected in duplicate together with CMV-βgal (200 ng/cm^2) to normalize for transfection efficiency. Twentyfour hours after transfection cells were treated with the indicated F-actin targeting drugs and harvested the day after.

Gene expression analysis. RNA extraction from cells was performed with RNeasy Mini Kit (QIAGEN) according to the manufacturer's instructions. For RNA extraction from skin samples, the epidermis was dissociated from the derma after overnight incubation with 0.05% Trypsin/EDTA at 4°C and subsequently processed with NucleoSpin RNA Kit (Macherey-Nagel) according to the manufacturer's instructions. Reverse transcription and qPCR were performed as previously described[1]. Real-time qPCR analyses were carried out with triplicate samplings of retrotranscribed cDNAs on the QuantStudio 5 Real-Time PCR System thermal cycler and analyzed with QuantStudio™ Design and Analysis Software (ThermoFisher). Expression levels were normalized to RPLP0[2] or Gapdh gene for qRT-PCR analysis on nHEK cells or epidermis samples respectively. Potential contamination of dermal tissue in epidermis samples was evaluated by qRT-PCR on Acta2 marker gene. Skin biopsies contaminated by dermal tissue were excluded from further analysis. Primers are listed in Supplementary Data 3.

Immunofluorescence and immunohistochemistry. Cells were fixed at room temperature with 4% PFA in PBS for 10 minutes and permeabilized with a solution of 0.3% Triton X-100 in PBS for 10 minutes. Coverslips were saturated with Blocking Buffer (Goat Serum 10% in PBS 0.1% Triton [PBSt]) for 45 minutes and incubated overnight at 4 °C with the primary antibody. Fixed cells were incubated in the dark 1.5 hours with the secondary antibody conjugated with a fluorophore, previously diluted in 2% Goat Serum in PBS. Slides were washed and mounted in ProLong Diamond antifade with DAPI (ThermoFisher). For immunofluorescence and immunohistochemistry on skin tissues, biopsies were fixed with PFA, paraffin-embedded and cut in 10 µm-thick sections. Sections were re-hydrated and antigen retrieval was performed by incubation in citrate buffer 0.01 M pH 6 at 95°C for 20 minutes. Slides were then permeabilized for 10 min at RT with PBS 1% Triton X-100. For immunofluorescence samples were subsequently processed as described above. For immunohistochemistry samples were processed with the ImmPRESS Excel Stainig Kit (MP-7601) and primary antibodies were in Antibody diluent from Dako (S080981-2). Primary and secondary antibodies with their working dilutions are listed in Supplementary Data 4. Confocal and bright field images were obtained with a Leica TCS SP5 equipped with a CCD camera. Bright field images of IHC-stained skin sections were acquired with a Leica DMR microscope equipped with a Leica DFC 480 camera.

Western Blot. Whole-cell lysates were obtained by sonication in lysis buffer (50 mM HEPES (pH 7.5), 100 mM NaCl, 50 mM KCl, 1% Triton-X100, 5% glycerol, 0.5% Np40, 2 mM $MgCl_2$ and protease and phosphatase inhibitors). The western blot procedure was carried out as previously described[3]. Uncropped images of immunoblots are shown in Supplementary Fig. 7. Primary and secondary antibodies with their working dilutions are listed in Supplementary Data 4.

Flow cytometry. Freshly trypsinized p3 passage keratinocytes were stained for 30 min at 4°C with a FITC-conjugated anti-β1 integrin antibody (CD2900-MEM-101A). The stained cells were rinsed twice, resuspended in Sorting Buffer (PBS/BSA 0.1%, 25 mM HEPES pH 7.0) analysed and sorted on a BD FACS Aria sorter (BD Biosciences). See Supplementary Data 4 for antibodies working dilutions.

Analysis of published microarray data. All microarray data were measured on Affymetrix arrays and have been downloaded from NCBI Gene Expression Omnibus (GEO, http://www.ncbi.nlm.nih.gov/geo/). The arrays for human skin Squamous Cell Carcinoma (SCC) cell lines was from GSE66359[4]. The arrays for mouse skin tumors was from GSE63967[5]; only samples labeled "dorsal skin" or "primary skin carcinoma" were used for subsequent analyses. Z-scores were calculated for each gene from the normalized gene expression data of each sample; gene signature scores were calculated for each sample by summing the Z-scores of each gene composing the signature. Yap-conserved signature was as in Ref [6]; the Notch signature was based on the expression levels of the following Notch target genes: *HES1*, *HES2*, *HES5*, *HEY1*, *HEY2* and *NOTCH3*. Data were visualized and analyzed using Prism (GraphPad).

SUPPLEMENTARY REFERENCES

1. Aragona, M. *et al.* A mechanical checkpoint controls multicellular growth through YAP/TAZ regulation by actin-processing factors. *Cell* **154**, 1047–1059 (2013).

2. Minner, F. & Poumay, Y. Candidate housekeeping genes require evaluation before their selection for studies of human epidermal keratinocytes. *J. Invest. Dermatol.* **129**, 770–773 (2009).

3. Dupont, S. *et al.* FAM/USP9x, a deubiquitinating enzyme essential for TGFbeta signaling, controls Smad4 monoubiquitination. *Cell* **136**, 123–135 (2009).

4. Farshchian, M. *et al.* EphB2 Promotes Progression of Cutaneous Squamous Cell Carcinoma. *J. Invest. Dermatol.* **135**, 1882–1892 (2015).

5. McCreery, M. Q. *et al.* Evolution of metastasis revealed by mutational landscapes of chemically induced skin cancers. *Nature Medicine* **21**, 1514–1520 (2015).

6. Cordenonsi, M. *et al.* The Hippo transducer TAZ confers cancer stem cell-related traits on breast cancer cells. *Cell* **147**, 759–772 (2011).
